# DeepAlloDriver: a deep learning-based strategy to predict cancer driver mutations

**DOI:** 10.1093/nar/gkad295

**Published:** 2023-04-20

**Authors:** Qianqian Song, Mingyu Li, Qian Li, Xun Lu, Kun Song, Ziliang Zhang, Jiale Wei, Liang Zhang, Jiacheng Wei, Youqiong Ye, Jinyin Zha, Qiufen Zhang, Qiang Gao, Jiang Long, Xinyi Liu, Xuefeng Lu, Jian Zhang

**Affiliations:** State Key Laboratory of Medical Genomics, National Research Center for Translational Medicine at Shanghai, Ruijin Hospital, Shanghai Jiao Tong University School of Medicine, Shanghai, China; Medicinal Chemistry and Bioinformatics Center, Shanghai Jiao Tong University School of Medicine, Shanghai 200025, China; State Key Laboratory of Medical Genomics, National Research Center for Translational Medicine at Shanghai, Ruijin Hospital, Shanghai Jiao Tong University School of Medicine, Shanghai, China; Medicinal Chemistry and Bioinformatics Center, Shanghai Jiao Tong University School of Medicine, Shanghai 200025, China; State Key Laboratory of Medical Genomics, National Research Center for Translational Medicine at Shanghai, Ruijin Hospital, Shanghai Jiao Tong University School of Medicine, Shanghai, China; Medicinal Chemistry and Bioinformatics Center, Shanghai Jiao Tong University School of Medicine, Shanghai 200025, China; Nutshell Therapeutics, Shanghai 201210, China; Medicinal Chemistry and Bioinformatics Center, Shanghai Jiao Tong University School of Medicine, Shanghai 200025, China; Medicinal Chemistry and Bioinformatics Center, Shanghai Jiao Tong University School of Medicine, Shanghai 200025, China; Department of Assisted Reproduction, Shanghai Ninth People's Hospital, Shanghai Jiao Tong University School of Medicine, Shanghai 200011, China; Department of Biomedical Sciences, College of Veterinary Medicine and Life Sciences, City University of Hong Kong, Hong Kong 999077, China; Medicinal Chemistry and Bioinformatics Center, Shanghai Jiao Tong University School of Medicine, Shanghai 200025, China; Department of Assisted Reproduction, Shanghai Ninth People's Hospital, Shanghai Jiao Tong University School of Medicine, Shanghai 200011, China; State Key Laboratory of Medical Genomics, National Research Center for Translational Medicine at Shanghai, Ruijin Hospital, Shanghai Jiao Tong University School of Medicine, Shanghai, China; Medicinal Chemistry and Bioinformatics Center, Shanghai Jiao Tong University School of Medicine, Shanghai 200025, China; Medicinal Chemistry and Bioinformatics Center, Shanghai Jiao Tong University School of Medicine, Shanghai 200025, China; Liver Cancer Institute, Zhongshan Hospital, Key Laboratory of Carcinogenesis and Cancer Invasion (Ministry of Education), Fudan University, Shanghai, China; Department of Pancreatic Surgery, Shanghai General Hospital, Shanghai Jiao Tong University School of Medicine, Shanghai 200080, China; Medicinal Chemistry and Bioinformatics Center, Shanghai Jiao Tong University School of Medicine, Shanghai 200025, China; Department of Assisted Reproduction, Shanghai Ninth People's Hospital, Shanghai Jiao Tong University School of Medicine, Shanghai 200011, China; State Key Laboratory of Medical Genomics, National Research Center for Translational Medicine at Shanghai, Ruijin Hospital, Shanghai Jiao Tong University School of Medicine, Shanghai, China; Medicinal Chemistry and Bioinformatics Center, Shanghai Jiao Tong University School of Medicine, Shanghai 200025, China; School of Pharmaceutical Sciences, Zhengzhou University, Zhengzhou 450001, China

## Abstract

Driver mutations can contribute to the initial processes of cancer, and their identification is crucial for understanding tumorigenesis as well as for molecular drug discovery and development. Allostery regulates protein function away from the functional regions at an allosteric site. In addition to the known effects of mutations around functional sites, mutations at allosteric sites have been associated with protein structure, dynamics, and energy communication. As a result, identifying driver mutations at allosteric sites will be beneficial for deciphering the mechanisms of cancer and developing allosteric drugs. In this study, we provided a platform called DeepAlloDriver to predict driver mutations using a deep learning method that exhibited >93% accuracy and precision. Using this server, we found that a missense mutation in RRAS2 (Gln72 to Leu) might serve as an allosteric driver of tumorigenesis, revealing the mechanism of the mutation in knock-in mice and cancer patients. Overall, DeepAlloDriver would facilitate the elucidation of the mechanisms underlying cancer progression and help prioritize cancer therapeutic targets. The web server is freely available at: https://mdl.shsmu.edu.cn/DeepAlloDriver.

## Graphical Abstract

**Graphical Abstract:**
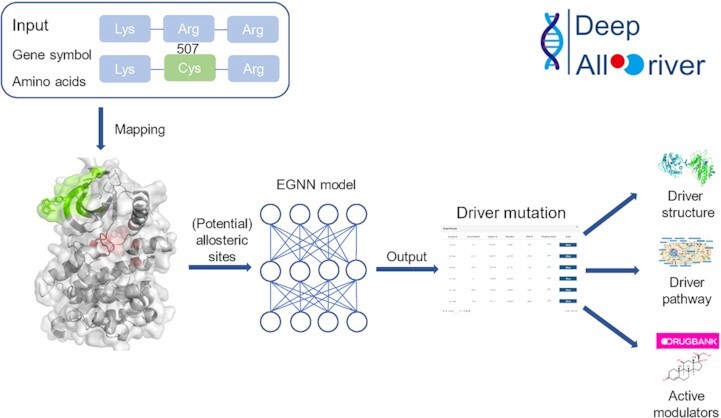
Identification of allosteric driver mutations in cancers.

## INTRODUCTION

Cancer is a genetic disease caused by mutations that directly or indirectly provide cancer cells with selective advantages for proliferation and invasion (1). The vast majority of mutations occurring in cancer cells are passengers, irrespective of the cancer phenotype and biological effect, while a small number of driver mutations confer cell growth and survival (2–4). A significant challenge in cancer therapy is distinguishing driver mutations from passenger mutations. With the development of whole genome and/or exome sequencing, the generation of resourceful databases, and machine/deep learning methods, several approaches, tools, and platforms have been proposed to identify driver mutations (5–14). However, a vital strategy for identifying driver mutations in the protein-coding regions is lacking.

Mutations in protein-coding regions are typically associated with altered biological functions. A small fraction of the mutations that occur at the catalytic sites has been extensively studied for their loss-of-function or gain-of-function effects, which are attributed to the disruption of the interaction between the natural ligand and protein (15–17). However, little attention has been paid to remote mutations that can also perturb protein function through allosteric regulation (15,16). Allosteric regulation plays a vital role in all cellular processes, including signal transduction, enzymatic catalysis, cellular metabolism, and gene regulation. From a structural perspective, allosteric mutations can induce a population shift and, therefore, destabilize or stabilize active or inactive states. Kurochkin *et al.* (17) demonstrated that some mutations in function-relevant distal sites resulted in an increase in the catalytic activity of the insulin-degrading enzyme by stabilizing its active state. Shen *et al.* (18) reported that deleterious mutations identified in cancer genomes were more significantly enriched at protein allosteric sites than tolerated mutations. Tee *et al.* (19) suggested that SNPs may function allosterically and that mutations at critical positions in the protein sequence can allosterically disrupt protein function. Additionally, targeting mutants at allosteric sites offers various advantages for drug discovery, including enhanced drug selectivity and efficacy compared to those at buried active/orthosteric sites. As a result, identifying allosteric driver mutations is critical for tumorigenesis and tumor-specific target discovery (20).

Based on previous studies (12,18), we proposed a deep learning method called DeepAlloDriver and offered a platform to identify allosteric driver mutations and annotate driver mutations, genes, and proteins. In the benchmarking test dataset, DeepAlloDriver detected driver mutations with >93% accuracy and precision. Furthermore, we employed DeepAlloDriver to identify an allosteric driver mutation in the Ras-related protein R-Ras2 (RRAS2) (Gln72 to Leu, RRAS2^Q72L^), which is supported by a knock-in model (21). Overall, DeepAlloDriver offers a new perspective for allosteric drug target design in addition to revealing the mechanisms of cancer.

## MATERIALS AND METHODS

### Workflow of DeepAlloDriver

DeepAlloDriver was developed as a platform to identify driver mutations at allosteric and potential allosteric sites. The web server is free for all users without a login.

**Figure 1.**
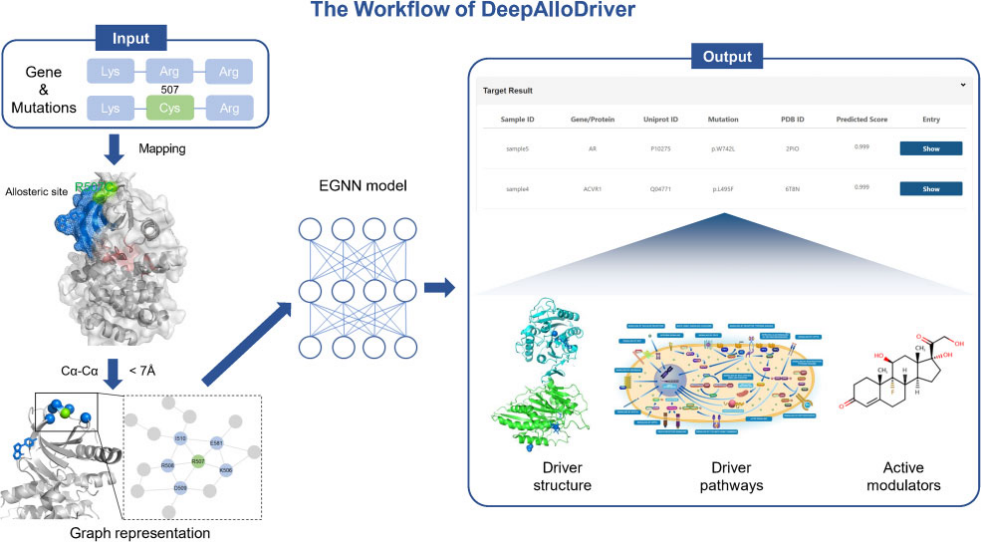
The Workflow of DeepAlloDriver.

The workflow of the DeepAlloDriver is shown in Figure 1. First, users can submit cancer samples, including gene symbols and amino acid substitutions. Substitutions are then extracted from samples and mapped to allosteric or potential allosteric sites derived from the RCSB Protein Data Bank (PDB) (22) and Allosteric Database (ASD) (23). Subsequently, the substitutions located at the mapped sites are evaluated for their potential driver effects using the server. In addition, the prediction score of the substitutions and location of the predicted substitutions on the protein structure, the corresponding gene or protein involved in the biological pathway, and modulators targeting proteins are displayed. Additional details of the workflow are provided in Supplementary Figure S1.

### Input

To offer greater flexibility, the web server allows users to upload three file formats. In the ‘Input’ text area, users can paste a valid amino acid substitutions list (each item is a substitution as follows: Simpleid;AR;G244D). Alternatively, users can upload cancer sample(s) in Mutation Annotation Format, which is a tab-delimited file containing somatic and/or germline mutation annotations. Moreover, uploading a tab-delimited file generated by ANNOVAR is permitted (24).

A ‘Job Name’ is needed, which helps users find their ‘Job Serial’ in the ‘Job Queue.’ DeepAlloDriver currently comprises 1949 proteins and 10 081 allosteric sites from ASD (http://mdl.shsmu.edu.cn/ASD) for the prediction of allosteric driver mutations. It is recommended that non-specialist users submit example tasks to master the server before uploading their samples. Example 1, containing six substitutions items, was tested for running time, and it took 30 seconds; users can input samples with <3600 mutation items according to their demands.

### Output

The output page consists of three parts. First, the ‘Job Queue’ bar lists the basic information about a job. Second, the middle ‘Job Progress’ section records the progress of calculations. Third, the ‘Target Result’ outputs the prediction result table once a job is finished. Mutations in the submitted sample would be ranked according to the ‘Predicted Score’ in the ‘Target Result’ table. The first six columns account for the protein and mutation information, such as ‘Sample ID’, ‘Gene/Protein’, ‘Uniprot ID’, ‘Mutation’, ‘PDB ID’ and ‘Predicted Score’. Furthermore, clicking the ‘Show’ button in each ‘Entry’ can direct the users to more detailed information about the mutation, and users can click the ‘Show’ button to navigate the details available.

The jump page pops up when users click the ‘Show’ button of the ‘Target Result’ table, displaying the annotation for the mutation and its protein; the detailed page contains ‘Target information’, ‘Pathway in the Reactome’ and ‘Known drugs on the target’. At the top of the detailed page, an interactive 3D representation powered by the JSMol plugin demonstrates the predicted substitution in the protein structure. The panel next to it exhibits some useful information like ‘Gene Symbol,’ ‘NCBI Gene ID’ with an internet link to the NCBI Gene database, ‘Function,’ ‘PDB ID,’ ‘Mutation’, and ‘Predicted Score’. The middle of the page illustrates the ‘Pathway in Reactome’ table summarizing the biological pathways in which the potential driver mutations were annotated from the Reactome pathway database (25). Particularly, clicking the ‘reactome’ button at the end of each entry allows visualization of the 2D diagram of signaling pathway. At the bottom, users can examine known modulators of the potential driver protein in the ‘Known drugs on the target’ table, which describes the compound name, the ID in DrugBank (26) or CHEMBL (27), the 2D structure, molecular weight, and the indication of the compound. A detailed explanation of pages is provided on the ‘Help’ page for user convenience.

## PERFORMANCE

We collected three datasets, including mutations from public databases (e.g. TCGA, ICGC and COSMIC), 17181 known driver mutations from various resources (e.g. CGI and IntOGen), and allosteric and potential allosteric sites from the PDB and ASD databases (detailed in the ‘Dataset Collections’ section and Supplementary Tables S1 and S2 in Supplementary Information). Of the 17 181 mutations located in allosteric sites, 8565 were defined as positive allosteric driver mutations, and the same number of passenger mutations at allosteric sites were obtained from these resources as negative allosteric passenger mutations. Additionally, 17 130 allosteric driver and passenger mutations were randomly split into the training set, validation set, and test set at a ratio of 8:1:1 (13 704 for the training set, 1713 for the validation set, and 1713 for the test set). In the training and test sets, 149 proteins and 1373 allosteric sites in these proteins were used according to the driver mutations (Supplementary Tables S3 and S4).

The classification power of DeepAlloDriver was benchmarked on a test set of 1713 mutants with optimized hyperparameters after 5-fold cross-validation (Supplementary Tables S5 and S6). The DeepAlloDriver score distributions of the allosteric driver and passenger mutations were built using an equivariant multi-head attention weighted graph neural network (EGNN) and a threshold of 0.5 can best separate the score distributions of the positives and negatives (Supplementary Figure S2). We used 0.5 as the threshold to obtain the performance scores, and mutations with >0.5 probability, were assigned as drivers. Our model detected allosteric driver mutations with 94.1% accuracy, 93.8% precision, 94.3% recall, 93.9% specificity and 94.1% F1 score (Supplementary Tables S5 and S6). Furthermore, we analyzed the classification power of our model via the receiver operating characteristic (ROC) curves (Supplementary Figure S2). The area under the ROC curve (AUC) was computed as 0.975, indicating the significant predictive power of DeepAlloDriver.

DeepAlloDriver was also compared with the previous AlloDriver server using the aforementioned blind test set containing 1713 allosteric mutations, as mentioned previously. As shown in Supplementary Table S7, DeepAlloDriver exhibited an average improvement of approximately 47% in predictive accuracy compared with AlloDriver, ranging from accuracy and precision to recall metrics. Additionally, it demonstrated a nearly 36% increase in the classification balance metrics, including specificity and the F1 score. Overall, DeepAlloDriver achieved a much higher the area under the receiver operating characteristic curve (AUROC) value, indicating its superior performance in classifying allosteric mutations. In general, a comparison between DeepAlloDriver and AlloDriver revealed that the performance of DeepAlloDriver was significantly better than that of AlloDriver, providing a useful tool for understanding the allosteric mechanisms of cancer progression.

## EXAMPLES

### Case 1: allosteric driver mutation on NTRK1

**Figure 2.**
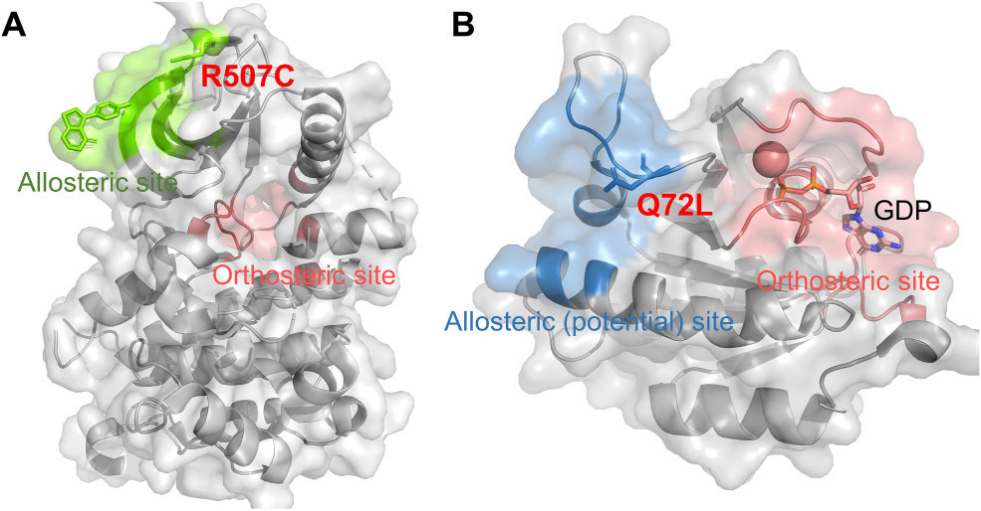
The examples of DeepAlloDriver. (**A**) The driver mutation R507C is found at the NTRK1 allosteric site (PDB ID: 6D20). The protein structure is exhibited in both cartoon and transparent surface modes with allosteric site coloured in chartreuse and orthosteric site in deepsalmon. Residue R507C is highlighted in stick mode. (**B**) The driver mutation Q72L is found at the RRAS2 potential allosteric site (PDB ID: 2ERY). Allosteric and orthosteric sites are highlighted in skyblue and deepsalmon, respectively. Residue Q72L and ligand GDP are shown in stick mode.

The neurotrophic receptor tyrosine kinase 1 (NTRK1, also known as MTC, TRK, TRK1, TRKA or Trk-A), is a membrane-bound receptor that phosphorylates itself and members of the mitogen-activated protein kinase (MAPK) pathway upon neurotrophin binding. In addition to the most common oncogenic NTRK fusions, NTRK mutations have been explored as potential oncogenic events (28,29). According to the server results, the mutation in Arg507 of the NTRK1 protein is located in an allosteric site composed of Arg507, Val511, Leu512, Lys513, Trp514, Lys523, Phe525 and Leu526, and the cysteine mutation on Arg507 (NTRK1^R507C^) could trigger allosteric communication with a score of 1.000 (Figure 2A), leading to a negative effect on the catalytic function of NTRK1. Output analyses have indicated that the region where Arg507 is located is also a hub of regulation across the protein kinase superfamily, and there are now 25 known type IV allosteric inhibitors or activators determined in the experimental structures bound to the region, destabilizing or strengthening the interactions with regulatory subunits (30). Overall, these findings indicate the possibility of using NTRK1^R507C^ as a cancer target and offer an active reference for the rational design of novel therapeutic agents.

### Case 2: allosteric driver mutation on RRAS2

The Ras-related protein R-Ras2 (RRAS2, also known as teratocarcinoma oncogene 21 (TC21)) is a Ras-like GTPase that shares downstream effectors with the Ras subfamily proteins. The abnormal function of RRAS2 is a triggering factor that perturbs downstream tumor signaling cascades (such as MEK-ERK signaling and the PI3K-mTOR pathway), promoting malignant transitions in various cancers (31,32). Using DeepAlloDriver, we found that RRAS2^Q72L^ could act as a strong driver mutation (output score = 0.995) by inducing internal conformational regulation from an allosteric site (Gln72, Glu73, Glu74, Phe75, Gln81, Met83, Arg84, Gln110 and Arg112) (Figure 2B), providing a mechanism for the mutation as a potent oncogenic driver in both RRAS2^Q72L^ knock-in mice and cancer patients (21).

## DISCUSSION

Allostery is a regulatory approach that transmits information in biological systems and can be utilized to decipher molecular mechanisms in a wide spectrum of biological processes and discover cancer driver proteins. DeepAlloDriver provides an efficient service to help clinicians and biologists with better decision-making in identifying allosteric driver mutations and carcinoma-relevant targets (30). Based on the results, the key factors contributing to the performance of DeepAlloDriver were investigated. First, DeepAlloDriver employs EGNNs, which are highly expressive and well suited for handling biomolecular graph data and capturing intricate patterns. Second, a larger dataset trained in DeepAlloDriver provided more diverse types of allosteric driver mutations, allowing the model to learn more complex patterns and better generalize to unseen data. Third, DeepAlloDriver uses raw data, specifically the 3D Cartesian coordinates of all Cα atoms in the protein, which enables the model to learn complex relationships and patterns directly from the data. To extend the scope of the server, 1949 proteins and 10 081 allosteric sites from ASD (http://mdl.shsmu.edu.cn/ASD) are provided for the prediction of allosteric driver mutations. Overall, DeepAlloDriver highlights the strong correlation between protein structure and function as well as the superior ability of EGNNs to predict allosteric driver mutations, and the effectiveness of the server was validated in the cancer drivers RRAS2 and NTRK1. This study has some limitations. It did not include driver mutation identification across more extensive disease types that are not currently associated with cancer. In addition, accumulative allosteric data incorporating allosteric modulators and proteins, together with the AlphaFold database (33), could allow for systematic profiling of allosteric sites in the human structural proteome. These improvements make DeepAlloDriver a more useful resource for the identification of allosteric driver mutations.

## DATA AVAILABILITY

DeepAlloDriver is freely available at: https://mdl.shsmu.edu.cn/DeepAlloDriver.

## Supplementary Material

gkad295_Supplemental_File
