# Supplementary material for: DeepAlloDriver: a deep learning-based strategy to predict cancer driver mutations
Source: Nucleic Acids Res. 2023 Apr 20;51(W1):W129–33. doi: 10.1093/nar/gkad295 (PMC10320081; doi:10.1093/nar/gkad295)
Supplement: gkad295_Supplemental_File [file gkad295_supplemental_file.pdf]

Supplementary Information

DeepAlloDriver: a deep learning-based web strategy to predict cancer driver mutations

Qianqian Song[1,2,†], Mingyu Li[1,†], Qian Li[2,†], Xun Lu[1,2,†], Kun Song[3], Ziliang Zhang[2], Jiale Wei[2,4], Liang Zhang[5], Jiacheng Wei[2,4], Youqiong Ye[1], Jinyin Zha[2], Qiufen Zhang[2], Qiang Gao[6], Jiang Long[7,*], Xinyi Liu[2,*], Xuefeng Lu[4,*], Jian Zhang[1,2,8,*]

[1] State Key Laboratory of Medical Genomics, National Research Center for Translational Medicine at Shanghai, Ruijin Hospital, Shanghai Jiao Tong University School of Medicine, Shanghai, China.

[2] Medicinal Chemistry and Bioinformatics Center, Shanghai Jiao Tong University School of Medicine, Shanghai, 200025, China.

[3] Nutshell Therapeutics, Shanghai, 201210, China.

[4] Department of Assisted Reproduction, Shanghai Ninth People's Hospital, Shanghai Jiao Tong University School of Medicine, Shanghai, 200011, China.

[5] Department of Biomedical Sciences, College of Veterinary Medicine and Life Sciences, City University of Hong Kong, Hong Kong, 999077, China.

[6] Liver Cancer Institute, Zhongshan Hospital, Key Laboratory of Carcinogenesis and Cancer Invasion (Ministry of Education), Fudan University, Shanghai, China

[7] Department of Pancreatic Surgery, Shanghai General Hospital, Shanghai Jiao Tong University School of Medicine, Shanghai 200080, China.

[8] School of Pharmaceutical Sciences, Zhengzhou University, Zhengzhou, 450001, China.

Corresponding Author

Jian Zhang
Email: Jian.zhang@sjtu.edu.cn
Tel: +86-21-63846590
Fax: +86-21-64154900

Xuefeng Lu
Email: xuefenglu163@163.com
Tel: +86-21-63846590
Fax: +86-21-64154900

Xinyi Liu
Email: coolist_liu@126.com
Tel: +86-21-63846590
Fax: +86-21-64154900

Jiang Long

Email: jiang.long@shgh.cn
Tel: +86-21-63240090
Fax: +86-21-63240090

† The authors wish it to be known that, in their opinion, the first 4 authors should be regarded as joint First Authors.

Contents

1. Dataset Collections 4

    1.1 Known Driver Gene Mutations 4

    1.2 Missense Mutations 4

    1.3 Allosteric Sites and Potential Allosteric Sites 4

    1.4 PDB Files Download 4

2. Material and Methods 5

    2.1 Model Construction 5

    2.2 Training Process 6

3. Tables 6

4. Figures 40

1. Dataset Collections

1.1 Known Driver Gene Mutations

We collected known driver gene mutations from Cancer Genome interpreter (CGI: https://www.cancergenomeinterpreter.org/2018/home;CGI_catalog_of_validated_oncogenic_mutations_20180117.csv) (1), driver genes from Integrative OncoGenomics (IntOGen: https://www.intogen.org/;https://www.intogen.org/download?file=IntOGen-Drivers-20200201.zip) (2), accepted evidence mutations from Clinical Interpretation of Variants in Cancer (CIViC: https://civicdb.org/downloads/01-Jul-2022/01-Jul-2022-ClinicalEvidenceSummaries.tsv) (3), highly curated known, disease-causing mutations from Database of Curated Mutations (DoCM version 3.2: http://www.docm.info/;variants.tsv) (4), FDA-recognized genetic variant from MSK's precision Oncology Knowledge Base (OncoKB: https://www.oncokb.org/;cancerGeneList.tsv) (5), and cancer variants from Precision Medicine Knowledgebase (PMKB: https://pmkb.weill.cornell.edu/therapies/download.csv; https://pmkb.weill.cornell.edu/variants/download.csv) (6). Then, we integrated and filtered the data and got 715 driver genes with 17,181 mutations.

1.2 Missense Mutations

We download somatic mutations from TCGA (hg19, 33 cancer types) (7), ICGC (GRCh37, 50 types cancers projects) (8), as well as COSMIC (v94, CosmicMutationExportCensu_GRCh37.tsv) (9). Next, we extracted missense mutations (non-synonymous mutations) items only.

1.3 Allosteric Sites and Potential Allosteric Sites

We manually collected and labeled the active sites binding by ligands and allosteric sites targeted by modulators of human proteins, and retrieved the pockets within 8 Å distance from protein ligands or modulators by PyMOL(10). We also included potential allosteric sites which scores greater than 0.6 in Allosteric Database (ASD) (11).

1.4 PDB Files Download

The 3D structure of proteins was retrieved from the RCSB Protein Data Bank (PDB, https://www.rcsb.org/) (12). We used a summary table processed by The Structure Integration

with Function, Taxonomy and Sequences resource (SIFTS, https://www.ebi.ac.uk/pdbe/docs/sifts/quick.html, pdb_chain_uniprot.tsv.gz) (13) to obtain a one-on-one mapping between PDB chains and UniProt (14) accession numbers.

Of the 17,181 mutations located in allosteric sites, 8,565 were defined as positive allosteric driver mutations, and the same number of passenger mutations at allosteric sites were obtained from these resources as negative allosteric passenger mutations. Additionally, 17,130 mutations of allosteric driver and passenger mutations were split into the training set, validation set, and test set by a ratio of 8:1:1 (13,704 for training set, 1,713 for validation set, and 1,713 for test set). Besides, in the training/test model, 149 proteins and 1,373 allosteric sites in these proteins were used according to known driver mutations and deposited in the Table S6 and S7.

2. Material and Methods

2.1 Model Construction

DeepAlloDriver detects allosteric driver mutations by discriminating whether a mutation situated in the allosteric (or potential allosteric) site is a driver or not utilizing and adapting equivariant transformer (ET) architecture in TorchMD-NET (15), an equivariant multi-head attention weighted graph neural networks (EGNNs) designed for efficiently handling graph data structure. The graph is the natural representation of biomolecules (atoms with covalent bonds connecting them) in the context of biochemistry, consisting of a set of nodes (i.e., atoms) and edges (i.e., bonds) connecting the nodes.

In reality, biomolecules could be transformed through inversion, rotation or translation, and these geometric symmetries have no effect on the physics of biomolecular systems. To truly portray the geometric symmetries of biomolecules, equivariant transformations are introduced in the ET. Moreover, a multi-head attention mechanism is applied to better integrate neighbouring atoms influence and local environment information in a biomolecular system (16) . As for proteins, simple but can capture the protein topology efficiently, we considered Cα atoms of other amino acids flanking the variant within a distance threshold (~7 Å) as protein context (17). Next, we defined the graph with variant and context Cα atoms as nodes, and edges drawn by Cα - Cα distance between the pair of nodes. Instead of selecting several conventional hand-

crafted representations, we featured each node with only a dimension of 40 vectors to represent wild type and variant amino acids via one-hot encoding, while the latter 20-dimensional vectors of context nodes were padded with null vectors. We adapted the ET with cross entropy loss function to make it better suited to the driver or not driver classification task, and we only extracted the latent representations of variant amino acids for the last loss computation. Finally, we used the sigmoid function to integrate the latent representations and return a possibility between 0 and 1.

2.2 Training Process

With the help of the distributed training protocol, we trained our models across 4 NVIDIA RTX 3080 Ti GPUs. Models were fine-tuned with 5-fold cross validation to obtain the optimal hyper-parameters. We used the AdamW optimization approach with a learning rate of 2e-4. And to properly adjust the learning rate whenever loss plateaus, we applied the reduce-on-plateau scheduler by setting the learning rate factor as 0.8 and a learning rate patience of 5. Models will wait for 30 epochs before an early stop if there is no progress on the validation set. Additionally, in table Table S3, we only presented the main hyperparameters that were changed and finally chose the bolded hyperparameters for better performance.

3. Tables

Table S1. Somatic mutation datasets from TCGA

Index	Projects	Cancer Information	Source
1	ACC-US	Adrenocortical Carcinoma	TCGA, US
2	BLCA-US	Bladder Urothelial Cancer	TCGA, US
3	BRCA-US	Breast Cancer	TCGA, US
4	CESC-US	Cervical Squamous Cell Carcinoma	TCGA, US
5	CHOL-US	Cholangiocarcinoma	TCGA, US
6	COAD-US	Colon Adenocarcinoma	TCGA, US
7	DLBC-US	Lymphoid Neoplasm Diffuse Large B-cell Lymphoma	TCGA, US
8	ESCA-US	Esophageal Carcinoma	TCGA, US

9	GBM-US	Brain Glioblastoma Multiforme	TCGA, US
10	HNSC-US	Head and Neck Squamous Cell Carcinoma	TCGA, US
11	KICH-US	Kidney Chromophobe	TCGA, US
12	KIRC-US	Kidney Renal Clear Cell Carcinoma	TCGA, US
13	KIRP-US	Kidney Renal Papillary Cell Carcinoma	TCGA, US
14	LAML-US	Acute Myeloid Leukemia	TCGA, US
15	LGG-US	Brain Lower Grade Glioma	TCGA, US
16	LIHC-US	Liver Hepatocellular carcinoma	TCGA, US
17	LUAD-US	Lung Adenocarcinoma	TCGA, US
18	LUSC-US	Lung Squamous Cell Carcinoma	TCGA, US
19	MESO-US	Mesothelioma	TCGA, US
20	OV-US	Ovarian Serous Cystadenocarcinoma	TCGA, US
21	PAAD-US	Pancreatic Cancer	TCGA, US
22	PCPG-US	Pheochromocytoma and Paraganglioma	TCGA, US
23	PRAD-US	Prostate Adenocarcinoma	TCGA, US
24	READ-US	Rectum Adenocarcinoma	TCGA, US
25	SARC-US	Sarcoma	TCGA, US
26	SKCM-US	Skin Cutaneous melanoma	TCGA, US
27	STAD-US	Gastric Adenocarcinoma	TCGA, US
28	TGCT-US	Testicular Germ Cell Tumors	TCGA, US
29	THCA-US	Head and Neck Thyroid Carcinoma	TCGA, US
30	THYM-US	Thymoma	TCGA, US
31	UCEC-US	Uterine Corpus Endometrial Carcinoma	TCGA, US
32	UCS-US	Uterine Carcinosarcoma	TCGA, US
33	UVM-US	Uveal Melanoma	TCGA, US

Table S2. Somatic mutation of cancer from the ICGC Data Portal Release 28

Index	Projects	Cancer Information	Source

1	ALL-US	Acute Lymphoblastic Leukemia	TARGET, US
2	AML-US	Acute Myeloid Leukemia	TARGET, US
3	BLCA-CN	Bladder Cancer	CN
4	BOCA-FR	Soft Tissue cancer- Ewing sarcoma	FR
5	BOCA-UK	Bone Cancer	UK
6	BPLL-FR	B-Cell Prolymphocytic Leukemia	FR
7	BRCA-UK	Breast Triple Negative/Lobular Cancer	UK
8	BTCA-JP	Biliary Tract Cancer	JP
9	BTCA-SG	Biliary Tract Cancer	SG
10	CLLE-ES	Chronic Lymphocytic Leukemia	ES
11	CMDI-UK	Chronic Myeloid Disorders	UK
12	COCA-CN	Colorectal Cancer	CN
13	EOPC-DE	Early Onset Prostate Cancer	DE
14	ESAD-UK	Esophageal Adenocarcinoma	UK
15	ESCA-CN	Esophageal Cancer	CN
16	GACA-CN	Gastric Cancer	CN
17	LAML-CN	Leukemia	CN
18	LAML-KR	Acute Myeloid Leukemia	KR
19	LIAD-FR	Benign Liver Tumour	FR
20	LICA-CN	Liver Cancer	CN
21	LICA-FR	Liver Cancer	FR
22	LIHM-FR	Liver Cancer - Hepatocellular macronodules	FR
23	LINC-JP	Liver Cancer-NCC	JP
24	LMS-FR	Soft tissue cancer - Leiomyosarcoma	FR
25	LUSC-CN	Lung Cancer	CN
26	LUSC-KR	Lung Cancer	KR
27	MALY-DE	Malignant Lymphoma	DE
28	MELA-AU	Skin Cancer	AU
29	NACA-CN	Nasopharyngeal cancer	CN

30	NBL-US	Neuroblastoma	TARGET, US
31	NKTL-SG	Blood Cancer - T-cell and NK-cell lymphoma	SG
32	ORCA-IN	IN Oral Cancer	IN
33	OV-AU	Ovarian Cancer	AU
34	PAEN-AU	Pancreatic Cancer Endocrine neoplasms	AU
35	PAEN-IT	Pancreatic Endocrine Neoplasms	IT
36	PBCA-DE	Pediatric Brain Cancer	DE
37	PBCA-US	Pediatric Brain Tumor - Multiple subtypes	CHOP, US
38	PEME-CA	Pediatric Medulloblastoma	CA
39	PRAD-CA	Prostate Adenocarcinoma	CA
40	PRAD-CN	Prostate Cancer	CN
41	PRAD-FR	Prostate Cancer - Adenocarcinoma	FR
42	PRAD-UK	Prostate Adenocarcinoma	UK
43	RECA-CN	Renal Cancer	CN
44	RECA-EU	Renal Cell Cancer	EUci/FR
45	RT-US	Rhabdoid Tumors	TARGET, US
46	SKCA-BR	Skin Adenocarcinoma	BR
47	THCA-CN	Thyroid Cancer	CN
48	THCA-SA	Thyroid Cancer	SA
49	UTCA-FR	Uterine Cancer - Carcinosarcoma	FR
50	WT-US	Wilms Tumor	TARGET, US

Table S3. The 149 proteins used in training/test of model.

Index	Uniport ID	Protein Name
1	Q06124	Tyrosine-protein phosphatase non-receptor type 11
2	P04637	Cellular tumor antigen p53
3	P10275	Androgen receptor
4	P01112	GTPase HRas

5	P01116	GTPase KRas
6	Q02750	Dual specificity mitogen-activated protein kinase kinase 1
7	P00533	Epiermal growth factor
8	Q14145	Kelch-like ECH-associated protein 1
9	P15056	Serine/threonine-protein kinase B-raf
10	Q07157	Tight junction protein ZO-1
11	P03372	Estrogen receptor
12	P14210	Hepatocyte growth factor
13	O75874	Isocitrate dehydrogenase
14	P08476	Inhibin beta A chain
15	P42336	Phosphatidylinositol 4,5-bisphosphate 3-kinase catalytic subunit alpha isoform
16	Q13485	Mothers against decapentaplegic homolog 4
17	P29317	Ephrin type-A receptor 2
18	P68400	Casein kinase II subunit alpha
19	Q9UKU7	Isobutyryl-CoA dehydrogenase, mitochondrial
20	Q06187	Tyrosine-protein kinase BTK
21	Q9H7Z6	Histone acetyltransferase KAT8
22	Q09472	Histone acetyltransferase p300
23	Q9UM73	ALK tyrosine kinase receptor
24	P07949	Proto-oncogene tyrosine-protein kinase receptor Ret
25	P52333	Tyrosine-protein kinase JAK3
26	O60674	Tyrosine-protein kinase JAK2
27	P31749	RAC-alpha serine/threonine-protein kinase
28	Q04771	Activin receptor type-1
29	P21802	Fibroblast growth factor receptor 2
30	O00571	ATP-dependent RNA helicase DDX3X
31	Q13451	Peptidyl-prolyl cis-trans isomerase FKBP5
32	O60784	Target of Myb1 membrane trafficking protein

33	P63000	Ras-related C3 botulinum toxin substrate 1
34	P42345	Serine/threonine-protein kinase mTOR
35	Q15303	Receptor tyrosine-protein kinase erbB-4
36	P37231	Peroxisome proliferator-activated receptor gamma
37	P21580	Tumor necrosis factor alpha-induced protein 3
38	P42684	Tyrosine-protein kinase ABL2
39	Q8IVH8	Mitogen-activated protein kinase kinase kinase kinase 3
40	P36888	Receptor-type tyrosine-protein kinase FLT3
41	P63092	Guanine nucleotide-binding protein G(s) subunit alpha isoforms short
42	P08922	Proto-oncogene tyrosine-protein kinase ROS
43	P00519	Tyrosine-protein kinase ABL1
44	P04629	High affinity nerve growth factor receptor
45	P40337	von Hippel-Lindau disease tumor suppressor
46	P08581	Hepatocyte growth factor receptor
47	P61586	Transforming protein RhoA
48	O43791	Speckle-type POZ protein
49	P21860	Receptor tyrosine-protein kinase erbB-3
50	Q6P2Q9	Pre-mRNA-processing-splicing factor 8
51	Q9NRY4	Rho GTPase-activating protein 35
52	Q9BYW2	Histone-lysine N-methyltransferase SETD2
53	P04040	Catalase
54	Q86U86	Protein polybromo-1
55	Q86X55	Histone-arginine methyltransferase CARM1
56	P43405	Tyrosine-protein kinase SYK
57	P60484	Phosphatidylinositol 3,4,5-trisphosphate 3-phosphatase and dual-specificity protein phosphatase PTEN
58	P63096	Guanine nucleotide-binding protein G(i) subunit alpha-1
59	P07333	Macrophage colony-stimulating factor 1 receptor
60	P19793	Retinoic acid receptor RXR-alpha

61	Q15047	Histone-lysine N-methyltransferase SETDB1
62	Q92793	CREB-binding protein
63	O14980	Exportin-1
64	P11142	Heat shock cognate 71 kDa protein
65	P46100	Transcriptional regulator ATRX
66	P09211	Glutathione S-transferase P
67	Q15796	Mothers against decapentaplegic homolog 2
68	Q9BZK7	F-box-like/WD repeat-containing protein TBL1XR1
69	P10721	Mast/stem cell growth factor receptor Kit
70	P04626	Receptor tyrosine-protein kinase erbB-2
71	P25440	Bromodomain-containing protein 2
72	Q9UIF8	Bromodomain adjacent to zinc finger domain protein 2B
73	P23458	Tyrosine-protein kinase JAK1
74	Q13131	5'-AMP-activated protein kinase catalytic subunit alpha-1
75	Q96L73	Histone-lysine N-methyltransferase, H3 lysine-36 specific
76	Q9UMX1	Suppressor of fused homolog
77	Q99835	Smoothened homolog
78	Q16531	DNA damage-binding protein 1
79	P12931	Proto-oncogene tyrosine-protein kinase Src
80	P06400	Retinoblastoma-associated protein
81	P31751	RAC-beta serine/threonine-protein kinase
82	P36897	TGF-beta receptor type-1
83	P22681	E3 ubiquitin-protein ligase CBL
84	Q16288	NT-3 growth factor receptor
85	P12830	Cadherin-1
86	P45985	Dual specificity mitogen-activated protein kinase kinase 4
87	P16671	Platelet glycoprotein 4
88	P35968	Vascular endothelial growth factor receptor 2
89	Q9UPY3	Endoribonuclease Dicer

90	P51532	Transcription activator BRG1
91	O00255	Menin
92	Q9BZ95	Histone-lysine N-methyltransferase NSD3
93	P01111	GTPase NRas
94	Q9NZJ5	Eukaryotic translation initiation factor 2-alpha kinase 3
95	P25054	Adenomatous polyposis coli protein
96	Q9NUX5	Protection of telomeres protein 1
97	Q8WWN8	Arf-GAP with Rho-GAP domain, ANK repeat and PH domain-containing protein 3
98	P21359	Neurofibromin
99	P16234	Platelet-derived growth factor receptor alpha
100	P43354	Nuclear receptor subfamily 4 group A member 2
101	Q14839	Chromodomain-helicase-DNA-binding protein 4
102	Q8IWS0	PHD finger protein 6
103	P61769	Beta-2-microglobulin
104	P40692	DNA mismatch repair protein Mlh1
105	P36507	Dual specificity mitogen-activated protein kinase kinase 2
106	P08238	Heat shock protein HSP 90-beta
107	Q07889	Son of sevenless homolog 1
108	P06401	Progesterone receptor
109	P54764	Ephrin type-A receptor 4
110	P27037	Activin receptor type-2A
111	O14672	Disintegrin and metalloproteinase domain-containing protein 10
112	P29466	Caspase-1
113	P49902	Cytosolic purine 5'-nucleotidase
114	P02751	Fibronectin
115	P11362	Fibroblast growth factor receptor 1
116	Q9NYV4	Cyclin-dependent kinase 12
117	P22455	Fibroblast growth factor receptor 4

118	P27986	Phosphatidylinositol 3-kinase regulatory subunit alpha
119	A5YKK6	CCR4-NOT transcription complex subunit 1
120	Q05209	Tyrosine-protein phosphatase non-receptor type 12
121	Q06787	Fragile X messenger ribonucleoprotein 1
122	Q9Y6D9	Mitotic spindle assembly checkpoint protein MAD1
123	P07992	DNA excision repair protein ERCC-1
124	Q14790	Caspase-8
125	Q04721	Neurogenic locus notch homolog protein 2
126	P49959	Double-strand break repair protein MRE11
127	P51114	RNA-binding protein FXR1
128	P27797	Calreticulin
129	P81274	G-protein-signaling modulator 2
130	O75581	Low-density lipoprotein receptor-related protein 6
131	Q9UIQ6	Leucyl-cystinyl aminopeptidase
132	P07954	Fumarate hydratase, mitochondrial
133	P11309	Serine/threonine-protein kinase pim-1
134	P35222	Catenin beta-1
135	Q86YC2	Partner and localizer of BRCA2
136	P08253	72 kDa type IV collagenase
137	P34896	Serine hydroxymethyltransferase, cytosolic
138	Q07812	Apoptosis regulator BAX
139	O15550	Lysine-specific demethylase 6A
140	P31939	Bifunctional purine biosynthesis protein ATIC
141	Q9UNN5	FAS-associated factor 1
142	O95714	E3 ubiquitin-protein ligase HERC2
143	Q7KZI7	Serine/threonine-protein kinase MARK2
144	P46531	Neurogenic locus notch homolog protein 1
145	Q16832	Discoidin domain-containing receptor 2
146	Q9NR48	Histone-lysine N-methyltransferase ASH1L

147	Q9Y6K1	DNA (cytosine-5)-methyltransferase 3A
148	Q96QB1	Rho GTPase-activating protein 7
149	P43246	DNA mismatch repair protein Msh2

Table S4. The 1373 allosteric sites used in training/testing of model.

Index	Allosteric site[a]	Index	Allosteric site
1	Q06124_6cms_1_0.767	688	P23458_4ei4_1_0.675
2	Q06124_6cms_2_0.642	689	P23458_4ei4_2_0.721
3	P04637_5mcv_3_0.882	690	P61586_1dpf_1_0.692
4	P04637_5mcv_2_0.846	691	P10275_4oez_2_0.771
5	P10275_2pkl_2_0.716	692	P07949_5fm2_2_0.777
6	P10275_2pkl_1_0.629	693	P51532_5ea1_1_0.645
7	P01112_421p_1_0.839	694	P00519_3qrk_1_0.801
8	P04637_2j1y_6_0.833	695	Q09472_4pzr_1_0.759
9	P04637_2j1y_16_0.726	696	O60674_3tjd_2_0.782
10	P04637_1ycs_1_0.69	697	P63000_2p2l_2_0.8
11	P04637_1ycs_2_0.631	698	P25440_4mr6_1_0.847
12	P04637_7dhz_2_0.8	699	MAP2K1_Q02750_1s9j_A_1001_BBM
13	P04637_7dhz_1_0.841	700	Q02750_1s9j_1_0.743
14	P04637_7dhz_3_0.644	701	O00255_4gq3_2_0.631
15	P01116_4nmm_1_0.691	702	O00255_4gq3_1_0.697
16	P04637_5ecg_2_0.727	703	P15056_3pri_12_0.674
17	P04637_5ecg_4_0.622	704	P21802_2pvy_1_0.836
18	Q02750_4ark_1_0.812	705	P21802_2pvy_3_0.646
19	P01112_3rs2_1_0.659	706	P03372_4iu7_1_0.638
20	P04637_2bim_1_0.703	707	Q06124_6bmy_1_0.618
21	P04637_2bim_3_0.629	708	JAK2_O60674_5ut5_A_905_2HB
22	P00533_5yu9_2_0.675	709	P09211_1px7_1_0.729

23	Q14145_4ifj_2_0.72	710	P09211_1px7_2_0.62
24	P00533_1xkk_1_0.803	711	Q9BZ95_5upd_1_0.784
25	P15056_3ii5_1_0.652	712	NRAS_P01111_6ziz_A_203_EZZ
26	P01112_1jah_1_0.87	713	Q9NZJ5_4x7j_1_0.807
27	P04637_5mg7_1_0.624	714	P21802_2py3_1_0.725
28	P04637_5mg7_3_0.665	715	P25054_3nmz_2_0.714
29	P04637_5mg7_7_0.657	716	P25054_3nmz_3_0.683
30	P00533_5em7_1_0.715	717	P25054_3nmz_1_0.828
31	P00533_5em7_2_0.607	718	P29317_4trl_1_0.788
32	P01116_6b0y_1_0.84	719	P29317_4trl_2_0.635
33	P15056_5hid_3_0.712	720	P03372_1yim_1_0.775
34	P15056_5hid_1_0.817	721	P03372_1yim_2_0.605
35	P15056_3q96_2_0.699	722	Q9NUX5_5h65_1_0.823
36	P15056_3q96_3_0.664	723	P68400_3axw_1_0.609
37	P01116_4lpk_1_0.828	724	O60674_4p7e_1_0.766
38	P01116_4lpk_3_0.625	725	P63000_5qqk_1_0.728
39	P04637_2j1x_2_0.669	726	P63000_5qqk_4_0.69
40	P04637_2j1x_1_0.674	727	P00519_3qri_2_0.737
41	Q07157_4oep_1_0.859	728	P03372_5w9d_1_0.639
42	P15056_4ehe_1_0.608	729	P23458_4i5c_2_0.666
43	P01116_5oco_3_0.686	730	P23458_4i5c_1_0.63
44	P01116_5oco_6_0.636	731	Q04771_4c02_3_0.615
45	P04637_2j1y_1_0.792	732	P04629_5kmn_1_0.7
46	P04637_2j1y_3_0.624	733	P04629_5kmn_2_0.71
47	P04637_2j1y_2_0.797	734	P03372_5dz1_2_0.693
48	P01116_5vq6_1_0.808	735	Q8WWN8_5jd0_1_0.867
49	P01116_5vq6_3_0.841	736	Q8WWN8_5jd0_2_0.88
50	P03372_5e0w_1_0.667	737	P03372_4ivw_1_0.743
51	P14210_4o3u_1_0.708	738	P37231_3pba_1_0.639

52	P04637_5aba_2_0.71	739	P37231_1nyx_1_0.655
53	P04637_5aba_1_0.628	740	P31751_3d0e_2_0.82
54	O75874_4umx_1_0.658	741	P08581_5dg5_1_0.656
55	P15056_1uwj_1_0.7	742	Q04771_5oxg_1_0.612
56	P08476_2p6a_4_0.864	743	P52333_4qt1_1_0.741
57	P08476_2p6a_2_0.796	744	ABL2_P42684_3gvu_A_1002_STI
58	PIK3CA_P42336_5sx8_A_1101_71M	745	P21359_3p7z_2_0.671
59	PIK3CA_P42336_5sx8_A_1102_LUZ	746	P21359_3p7z_3_0.699
60	P15056_4mnf_1_0.683	747	P10275_2pix_2_0.764
61	Q13485_1g88_1_0.71	748	P10275_2pix_1_0.631
62	Q13485_1g88_2_0.897	749	P36888_5x02_1_0.791
63	EGFR_P00533_5d41_A_1103_57N	750	O60674_3jy9_1_0.832
64	P01116_4ept_1_0.786	751	P42345_4jsv_2_0.632
65	P01112_2quz_1_0.736	752	Q86X55_2y1w_4_0.624
66	P01116_4luc_2_0.755	753	Q86X55_2y1w_1_0.817
67	P01116_4luc_1_0.657	754	P16234_5grn_1_0.877
68	P29317_1mqb_1_0.661	755	Q02750_3dy7_1_0.649
69	P01112_2rgg_1_0.803	756	P08922_4uxl_1_0.738
70	P00533_5c8m_1_0.858	757	P00533_2j5f_3_0.675
71	P68400_4kwp_1_0.697	758	P03372_5dz1_1_0.688
72	P04637_5o1i_1_0.637	759	P09211_5l6x_1_0.734
73	P15056_1uwh_2_0.603	760	P23458_4ivb_1_0.725
74	P15056_1uwh_1_0.67	761	P42336_4l1b_4_0.629
75	P15056_5jsm_2_0.863	762	P03372_6chw_1_0.799
76	P15056_5jsm_1_0.861	763	P03372_5dxr_2_0.695
77	P01116_5uk9_1_0.865	764	P11142_4h5t_1_0.672
78	P04637_2j1y_8_0.881	765	P43354_1ovl_1_0.687

79	P04637_5aom_1_0.702	766	P43354_1ovl_4_0.673
80	P15056_3pri_1_0.732	767	P14210_5cs5_1_0.67
81	P15056_3pri_2_0.735	768	P00519_2hz0_2_0.743
82	P01116_5vq8_2_0.792	769	P08581_3zze_2_0.723
83	Q9UKU7_1rx0_1_0.697	770	P63000_5qqk_2_0.672
84	Q06187_5jrs_3_0.689	771	P52333_5lwm_1_0.609
85	Q06187_5jrs_4_0.69	772	P68400_4gub_1_0.834
86	Q9H7Z6_4dnc_2_0.716	773	NTRK1_P04629_6d20_A_801_FQG
87	P01116_5vq8_1_0.783	774	P31751_1o6l_3_0.688
88	P68400_4grb_1_0.662	775	P31751_1o6l_2_0.693
89	P03372_5dy8_2_0.646	776	P03372_1xpc_1_0.69
90	Q9H7Z6_3qah_1_0.695	777	P03372_5tmw_1_0.655
91	P01116_4tqa_1_0.695	778	P08476_2arv_1_0.795
92	P01116_4tqa_2_0.615	779	O60674_5tq5_1_0.821
93	P04637_1uol_1_0.796	780	P04040_1dgf_4_0.749
94	P15056_4g9c_1_0.798	781	P04040_1dgf_12_0.681
95	P15056_4g9c_2_0.736	782	P03372_5tmw_2_0.704
96	P01112_5wdp_1_0.732	783	Q14145_4n1b_8_0.602
97	P00533_4jr3_1_0.715	784	P10275_2ax6_2_0.807
98	Q09472_4pzs_1_0.85	785	P00533_4i23_2_0.614
99	Q09472_5lkx_1_0.705	786	P10275_2pnu_1_0.874
100	Q9UM73_4fnz_1_0.696	787	P68400_4fbx_1_0.659
101	O75874_4umx_9_0.617	788	Q14839_4o9i_1_0.828
102	O75874_4umx_3_0.827	789	Q14839_4o9i_2_0.648
103	P15056_3ii5_3_0.627	790	P21802_2pvy_5_0.745
104	P15056_4g9r_1_0.806	791	P15056_3pri_11_0.681
105	P15056_4g9r_2_0.769	792	P29317_5ia0_1_0.793
106	P00533_1mox_7_0.653	793	O00255_4gpq_1_0.668

107	P10275_4ofu_1_0.813	794	P03372_4iwc_1_0.74
108	P10275_4ofu_2_0.71	795	P37231_4hee_1_0.887
109	P68400_1pjk_1_0.71	796	P37231_3po9_1_0.628
110	P07949_5amn_1_0.838	797	P10275_2ax7_1_0.611
111	P04637_4agp_3_0.683	798	Q8IWS0_4nn2_2_0.618
112	P04637_4agp_1_0.825	799	Q8IWS0_4nn2_1_0.854
113	P52333_6aak_2_0.613	800	P61769_4ra3_3_0.753
114	P04637_5aoi_3_0.643	801	P61769_4ra3_1_0.684
115	P04637_5aoi_2_0.626	802	P12931_1fmk_1_0.737
116	P01112_3rs3_1_0.716	803	Q04771_5s87_3_0.682
117	P01112_5x9s_1_0.682	804	P09211_3pgt_1_0.701
118	P04637_2vuk_2_0.699	805	P09211_3pgt_3_0.679
119	P52333_1yvj_1_0.848	806	P40692_4p7a_1_0.664
120	P04637_5ab9_1_0.678	807	Q9BYW2_5lsy_3_0.722
121	P04637_5ab9_2_0.64	808	P14210_5cs5_2_0.703
122	Q02750_3eqf_2_0.694	809	P10275_3b67_1_0.892
123	P15056_5jrq_2_0.66	810	Q9H7Z6_4dnc_4_0.659
124	P15056_5jrq_1_0.635	811	P29317_5i9x_1_0.651
125	P01116_5oco_2_0.659	812	P36897_5qu0_1_0.871
126	P04637_7dhz_9_0.62	813	P68400_5m44_1_0.834
127	Q09472_3biy_1_0.802	814	P10275_1t65_2_0.759
128	P01112_3rs5_1_0.693	815	MAP2K2_P36507_1s9i_A_1001_5EA
129	PTPN11_Q06124_6bmu_A_602_DZV	816	Q13451_4dro_1_0.701
130	PTPN11_Q06124_6bmu_A_601_5OD	817	P09211_2a2r_1_0.825
131	O60674_4d1s_1_0.795	818	P68400_6a1c_1_0.791
132	P15056_5c9c_1_0.747	819	P15056_3prf_1_0.616
133	P15056_5c9c_2_0.854	820	O60674_5wev_1_0.854

134	P01112_1p2u_1_0.776	821	P03372_1xp6_1_0.795
135	P42336_4l1b_1_0.625	822	P35968_3b8r_1_0.696
136	P00533_2gs7_1_0.827	823	P03372_6chw_2_0.649
137	P00533_2gs7_2_0.771	824	P12830_4zte_1_0.862
138	P01116_5usj_4_0.655	825	P08238_3pry_6_0.783
139	P01116_5usj_1_0.676	826	P08238_3pry_5_0.691
140	P04637_5a7b_2_0.641	827	P00533_3w32_1_0.879
141	P15056_4cqe_1_0.86	828	Q07889_3ksy_1_0.742
142	P00533_5fed_2_0.816	829	P37231_3ia6_2_0.697
143	P01112_1wq1_1_0.747	830	Q13485_1g88_8_0.743
144	P10275_2ylo_1_0.89	831	P37231_3osi_1_0.652
145	P15056_4ehg_2_0.601	832	P63096_2xns_2_0.88
146	P31749_3qkl_1_0.72	833	P63096_2xns_1_0.676
147	Q06187_4otq_1_0.734	834	P08581_3f82_1_0.791
148	Q06187_4otq_2_0.647	835	P08476_2p6a_3_0.76
149	Q09472_5lku_1_0.644	836	Q13451_4drk_2_0.71
150	P15056_6cad_1_0.729	837	Q13451_4drk_1_0.698
151	P07949_2x2k_1_0.834	838	P04629_4pms_1_0.789
152	P42336_4l23_2_0.718	839	Q6P2Q9_4jkg_2_0.698
153	P42336_4l23_1_0.691	840	Q6P2Q9_4jkg_5_0.623
154	P15056_4ksq_2_0.615	841	Q9UKU7_1rx0_3_0.812
155	P15056_4ksq_1_0.63	842	P00533_5uwd_1_0.738
156	Q9UM73_4ans_1_0.764	843	P37231_3wmh_1_0.795
157	Q04771_5s83_2_0.731	844	P37231_3t03_1_0.841
158	P03372_5w9d_3_0.77	845	P03372_5kcd_2_0.732
159	P15056_6cad_2_0.787	846	O60674_5tq4_2_0.646
160	P21802_2pzp_1_0.691	847	P06401_4oar_2_0.807
161	P21802_2pzp_3_0.601	848	P06401_4oar_1_0.657
162	Q04771_5s7s_1_0.691	849	P23458_6c7y_1_0.69

163	O00571_5e7j_1_0.642	850	O60674_4f08_1_0.675
164	P04637_5o1b_1_0.643	851	O60674_4f08_2_0.722
165	P15056_5jsm_4_0.85	852	O60674_5ut1_1_0.852
166	P15056_5jsm_6_0.797	853	P54764_2wo1_1_0.729
167	P01112_5b30_1_0.792	854	P04629_4pmp_1_0.76
168	P15056_5jsm_3_0.713	855	P37231_4fgy_1_0.812
169	P01112_3oiw_1_0.693	856	P37231_4fgy_2_0.774
170	P04637_3q01_2_0.658	857	P21802_3cu1_1_0.765
171	P04637_3q01_3_0.635	858	P10275_2piu_2_0.757
172	P00533_5zwj_1_0.855	859	P10275_2piu_1_0.809
173	Q13451_4w9p_1_0.886	860	P27037_3q4t_1_0.803
174	P10275_2piq_1_0.759	861	P27037_3q4t_2_0.857
175	P10275_2piq_2_0.692	862	P36897_3hmm_1_0.895
176	O60784_1elk_2_0.623	863	P12931_4hxj_3_0.675
177	P03372_5kro_2_0.62	864	P12931_4hxj_2_0.7
178	O75874_5yfn_3_0.676	865	O14672_6be6_4_0.642
179	O75874_5yfn_6_0.658	866	P10275_4oed_2_0.773
180	Q02750_3vvh_1_0.672	867	P10275_4oed_1_0.765
181	P03372_5e0x_1_0.642	868	O75874_1t09_3_0.742
182	P03372_5e0x_2_0.647	869	O75874_1t09_2_0.675
183	P00533_2rgp_1_0.831	870	P04629_5kmj_2_0.62
184	P00533_3w33_1_0.821	871	P04629_5kmj_1_0.869
185	P01116_4obe_1_0.719	872	P37231_5dvc_1_0.703
186	P01112_1qra_1_0.852	873	P09211_3kmo_1_0.672
187	P04637_2j1y_7_0.643	874	O60674_4aqc_1_0.761
188	P00533_4tks_2_0.653	875	O60674_4aqc_2_0.709
189	P00533_4tks_1_0.757	876	O60674_4bbe_1_0.654
190	P04637_5mcv_1_0.887	877	P29466_2h4y_2_0.796
191	P04637_5o1f_2_0.687	878	P29466_2h4y_1_0.711

192	P04637_3d09_2_0.636	879	P35968_2qu6_2_0.821
193	P01116_6asa_1_0.704	880	P03372_5ak2_2_0.607
194	Q02750_3zm4_1_0.808	881	P03372_5ak2_1_0.676
195	P63000_5n6o_2_0.617	882	P10275_4oez_1_0.802
196	P63000_5n6o_4_0.608	883	NT5C2_P49902_2jc9_A_1497_ADN
197	P01112_3kkm_1_0.604	884	NT5C2_P49902_2jc9_A_1498_ADN
198	MTOR_P42345_1nsg_A_108_RAP	885	P14210_5cs3_2_0.725
199	P01116_5usj_2_0.729	886	P11142_3fzh_2_0.676
200	P10275_4oha_2_0.778	887	P08581_3qti_1_0.65
201	P10275_4oha_1_0.851	888	P08581_3qti_2_0.638
202	Q14145_3zgc_1_0.849	889	P43354_1ovl_2_0.664
203	Q14145_3zgc_4_0.65	890	CASP1_P29466_2fqq_B_1_F1G
204	P00533_5hcx_1_0.728	891	P23458_4e5w_1_0.773
205	P01116_5w22_1_0.714	892	P23458_4e5w_2_0.767
206	Q02750_3os3_1_0.771	893	P03372_5kcw_2_0.611
207	P01116_4epx_1_0.753	894	IDH1_O75874_5lge_B_502_6VN
208	P63000_2p2l_1_0.744	895	P68400_5h8e_3_0.719
209	P63000_2p2l_4_0.682	896	P68400_5h8e_1_0.629
210	Q15303_3bce_2_0.654	897	Q04771_5s7z_4_0.713
211	P00533_5hg9_2_0.76	898	Q04771_5s7z_1_0.831
212	P00533_5hg9_1_0.658	899	P61586_4xsh_3_0.617
213	P01112_2rgc_1_0.606	900	P61586_4xsh_4_0.705
214	P01112_3lbh_1_0.711	901	P02751_5dft_1_0.792
215	P37231_3lmp_1_0.824	902	P02751_5dft_2_0.732
216	P21580_3dkb_1_0.697	903	P11362_4rwi_3_0.665
217	Q9UM73_4anq_1_0.654	904	Q9NYV4_4nst_1_0.706
218	Q13485_1mr1_3_0.833	905	P22455_4tyg_1_0.63

219	P10275_3l3x_2_0.791	906	P22455_4tyg_3_0.749
220	P10275_3l3x_1_0.813	907	Q04771_5s7r_3_0.675
221	P00533_4i24_2_0.725	908	P36897_5qtz_1_0.887
222	P15056_4ehg_1_0.814	909	Q13451_4drm_1_0.687
223	P00533_5hib_1_0.653	910	P37231_3ty0_1_0.691
224	AKT1_P31749_4ejn_A_501_0R4	911	O75874_5l58_2_0.628
225	P31749_4ejn_1_0.733	912	O60674_4gmy_1_0.828
226	P10275_4olm_1_0.87	913	P08581_3cth_1_0.735
227	P10275_4olm_2_0.776	914	P03372_4xi3_3_0.626
228	P01116_5w22_6_0.698	915	P54764_3gxu_4_0.673
229	P04637_3d07_1_0.744	916	P68400_5h8e_2_0.662
230	Q09472_5lkz_1_0.792	917	P29317_3mbw_3_0.649
231	P42684_3hmi_1_0.834	918	P29317_3mbw_4_0.611
232	P15056_4fk3_2_0.797	919	P03372_2p15_2_0.736
233	P00533_1mox_1_0.828	920	P03372_2p15_4_0.654
234	PPARG_P37231_3k8s_B_2_Z27	921	Q15303_2r4b_2_0.628
235	Q13485_1mr1_2_0.692	922	P10275_2ax6_1_0.712
236	Q13485_1mr1_1_0.801	923	P09211_3kmo_2_0.688
237	Q8IVH8_5j5t_2_0.854	924	P10275_3b68_1_0.866
238	FLT3_P36888_4rt7_A_1001_P30	925	IDH1_O75874_6b0z_A_502_C81
239	P36888_4rt7_1_0.867	926	P27986_5gji_3_0.646
240	P63092_6au6_1_0.886	927	P27986_5gji_1_0.752
241	P29317_3czu_3_0.648	928	Q15047_6bhd_1_0.804
242	P29317_3czu_1_0.733	929	P00533_1mox_2_0.602
243	P03372_1yin_1_0.869	930	P37231_2f4b_1_0.67
244	Q14145_4l7d_1_0.778	931	P37231_2f4b_2_0.651
245	P08922_3zbf_1_0.726	932	Q04771_5s7r_1_0.674
246	P15056_4rzw_1_0.747	933	P06401_2ovh_1_0.646
247	Q04771_5s8a_2_0.7	934	Q06187_5bq0_1_0.65

248	Q04771_5s8a_1_0.65	935	Q06187_5bq0_3_0.622
249	P52333_3lxl_1_0.847	936	P08476_2p6a_1_0.792
250	P01112_3l8z_1_0.65	937	P03372_1xp1_1_0.714
251	P00533_4rj6_1_0.804	938	P01112_3lo5_2_0.603
252	P00519_5hu9_1_0.836	939	Q14145_5f72_1_0.862
253	P10275_1gs4_2_0.772	940	O43791_3hqi_6_0.737
254	P03372_4mga_3_0.674	941	P43405_4fl3_1_0.772
255	P01116_5w22_2_0.699	942	P37231_3b0r_1_0.621
256	P04629_4yne_1_0.833	943	P01112_1xcm_2_0.722
257	P10275_4oj9_2_0.699	944	P04040_1dgf_2_0.733
258	P04637_7dhz_4_0.634	945	Q9BYW2_5lt8_1_0.677
259	P42336_5xgh_2_0.665	946	Q9BYW2_5lt8_2_0.611
260	P40337_4wqo_5_0.679	947	AR_P10275_2pio_A_933_2MI
261	P00533_5cau_1_0.774	948	P10275_2pio_1_0.904
262	P03372_5aav_1_0.607	949	AR_P10275_2pio_A_934_2MI
263	P37231_3ia6_1_0.64	950	Q6P2Q9_3lru_2_0.747
264	P01116_5v6s_1_0.848	951	Q6P2Q9_3lru_3_0.63
265	KRAS_P01116_5v9o_A_203_91G	952	P10275_1z95_1_0.823
266	P00533_4li5_1_0.803	953	Q9UIF8_5orb_2_0.606
267	O60674_4e4m_3_0.674	954	Q9UIF8_5orb_1_0.655
268	O60674_4e4m_5_0.646	955	P27986_5gji_2_0.718
269	P01116_5oco_4_0.697	956	Q06124_4nwg_3_0.756
270	P04629_5kvt_1_0.8	957	Q06124_4nwg_4_0.746
271	P10275_4ok1_1_0.849	958	A5YKK6_4crv_3_0.66
272	P10275_4ok1_2_0.811	959	A5YKK6_4crv_5_0.672
273	O60674_4e4m_2_0.829	960	P68400_5m4c_1_0.798
274	P00533_4i1z_1_0.708	961	P63000_5n6o_1_0.722
275	P08581_3efk_1_0.778	962	P37231_4ci5_1_0.627

276	P08581_3efk_2_0.625	963	A5YKK6_4ct7_1_0.85
277	P61586_5fr1_1_0.64	964	Q14145_2flu_2_0.69
278	P01116_5f2e_1_0.866	965	P03372_5dz3_3_0.607
279	P01112_4dls_1_0.695	966	O60674_4e4m_1_0.65
280	P10275_5t8j_1_0.894	967	P31751_2xh5_2_0.787
281	P00533_5edr_1_0.837	968	P31751_2xh5_1_0.824
282	P15056_3pri_6_0.624	969	P01112_221p_2_0.633
283	O43791_3hqi_5_0.705	970	O60674_4zim_1_0.657
284	O43791_3hqi_1_0.762	971	O60674_4zim_3_0.748
285	P04637_5lap_2_0.651	972	P07949_2ivu_1_0.838
286	P04637_5lap_3_0.683	973	Q04771_5s7u_1_0.679
287	P15056_4ksp_4_0.608	974	IDH1_O75874_5sun_B_502_70Q
288	P04637_5mg7_6_0.753	975	P37231_3ho0_1_0.636
289	P04637_5mg7_2_0.816	976	O75874_4umx_10_0.626
290	P01116_4q03_1_0.703	977	O60674_4hge_1_0.717
291	Q06187_5bpy_7_0.649	978	O60674_4hge_5_0.634
292	P04637_4ago_2_0.73	979	P01112_5wpl_2_0.716
293	P04637_1kzy_1_0.715	980	P10275_3v4a_1_0.91
294	P10275_2ax9_1_0.75	981	Q05209_5j8r_1_0.825
295	P10275_2ax9_2_0.636	982	Q05209_5j8r_2_0.66
296	P01116_4epy_1_0.713	983	Q14145_4l7b_6_0.737
297	P00533_5ugc_2_0.786	984	O75874_4umx_4_0.677
298	Q9UKU7_1rx0_2_0.704	985	P00519_5mo4_1_0.838
299	P15056_5csx_1_0.764	986	Q06187_3p08_3_0.675
300	P03372_5dyd_3_0.716	987	P00519_3pyy_2_0.662
301	P01112_4dlt_1_0.71	988	P00519_3pyy_3_0.614
302	KRAS_P01116_4lv6_A_203_20H	989	Q9BYW2_5jle_2_0.636
303	P61586_4d0n_2_0.615	990	O43791_3hqi_4_0.624
304	P61586_4d0n_3_0.623	991	Q06787_2qnd_2_0.736

305	P01116_4dsn_1_0.712	992	Q06787_2qnd_5_0.775
306	P01112_3tgp_1_0.73	993	P07333_2i0y_1_0.82
307	O75874_3inm_1_0.608	994	Q06787_4qvz_3_0.631
308	P00533_5xdk_1_0.635	995	P08476_2b0u_2_0.699
309	P04637_4agl_2_0.705	996	Q9Y6D9_4dzo_1_0.735
310	P04637_4agl_3_0.657	997	Q9Y6D9_4dzo_3_0.614
311	P21860_1m6b_1_0.693	998	P07992_2a1i_1_0.823
312	Q02750_3v04_1_0.8	999	Q06187_4rx5_1_0.609
313	Q6P2Q9_4jk9_1_0.753	1000	P21802_2py3_2_0.721
314	Q6P2Q9_4jk9_3_0.756	1001	P37231_3et3_1_0.79
315	Q9UM73_4cmt_2_0.622	1002	P37231_3et3_2_0.793
316	P03372_5dks_2_0.622	1003	P08581_2wkm_1_0.745
317	Q9NRY4_3fk2_3_0.747	1004	O60674_6bbv_1_0.682
318	Q9BYW2_5lsx_1_0.645	1005	O60674_4fvr_2_0.706
319	P14210_5csq_2_0.666	1006	O60674_4fvr_1_0.669
320	P03372_4xi3_4_0.617	1007	P08581_3ccn_1_0.662
321	P10275_4okb_2_0.827	1008	Q06124_4h1o_1_0.687
322	P10275_4okb_1_0.851	1009	P11362_4rwl_1_0.644
323	P04040_1dgf_1_0.744	1010	P11362_4rwl_2_0.672
324	P04040_1dgf_11_0.684	1011	O60674_4e6q_3_0.607
325	P37231_2fvj_1_0.745	1012	O60674_5ut4_3_0.645
326	P08581_4xmo_1_0.811	1013	O60674_5ut4_1_0.881
327	P00533_5hg8_1_0.842	1014	Q04771_5s7q_1_0.672
328	P04637_1kzy_3_0.676	1015	Q04771_5s7q_2_0.636
329	P04637_3q01_1_0.682	1016	CASP8_Q14790_3kjn_A_900_DTT
330	P68400_2pvr_1_0.7	1017	Q14790_3kjn_1_0.67
331	P04040_1dgf_7_0.656	1018	P23458_5e1e_1_0.839
332	Q9UM73_4fod_1_0.882	1019	P23458_5e1e_2_0.752

333	P00533_2jiv_1_0.616	1020	P52333_5ttu_1_0.822
334	P01112_4dlw_1_0.722	1021	Q13451_3o5f_1_0.711
335	P37231_4xum_2_0.611	1022	P03372_2qse_2_0.702
336	P37231_4xum_1_0.671	1023	P25440_5dw1_2_0.657
337	P15056_5jsm_7_0.828	1024	P25440_5dw1_1_0.603
338	Q86U86_5fh6_3_0.769	1025	P11142_3m3z_1_0.79
339	Q86U86_5fh6_1_0.804	1026	P37231_5ugm_1_0.655
340	Q86X55_4ikp_1_0.618	1027	P68400_3c13_1_0.685
341	P43405_3fqh_1_0.671	1028	P03372_2q70_3_0.73
342	P43405_3fqh_2_0.758	1029	P37231_3adv_1_0.664
343	P10275_2yhd_1_0.645	1030	P61769_3vfw_3_0.785
344	P10275_2yhd_2_0.705	1031	P61769_3vfw_2_0.868
345	P04637_2j1y_5_0.671	1032	Q04721_2oo4_2_0.714
346	P01116_5oco_5_0.649	1033	Q15047_6bhg_1_0.742
347	P42336_4l23_6_0.822	1034	P22455_4qqj_1_0.673
348	P37231_5gto_1_0.762	1035	P22455_4qqj_3_0.691
349	P04637_5o1e_1_0.625	1036	P00519_3pyy_1_0.751
350	P01112_5b2z_1_0.722	1037	P36897_6b8y_1_0.705
351	Q04771_6eix_1_0.845	1038	P22455_4tye_3_0.628
352	P00533_4rj4_1_0.857	1039	P22455_4tye_1_0.767
353	P04637_2j1y_4_0.704	1040	P08476_2p6a_12_0.794
354	P01116_5vq6_8_0.672	1041	Q04771_5s7v_2_0.689
355	P15056_3og7_1_0.636	1042	Q04771_5s7v_3_0.823
356	O75874_5l58_1_0.809	1043	Q92793_4nyv_1_0.722
357	P60484_1d5r_1_0.632	1044	P14210_4o3t_1_0.73
358	P37231_4jl4_1_0.667	1045	P03372_5w9d_2_0.78
359	P63096_2gtp_2_0.65	1046	Q04771_5s83_3_0.812
360	P63096_2gtp_1_0.705	1047	P00533_4r5s_8_0.617
361	P00533_2itt_1_0.678	1048	P21580_3dkb_2_0.691

362	P07333_2i1m_1_0.655	1049	P68400_3wow_1_0.811
363	P07333_2i1m_2_0.858	1050	P21802_4j96_1_0.815
364	P01112_4efl_1_0.637	1051	Q9UM73_2xb7_2_0.611
365	P01116_4lyf_1_0.845	1052	P02751_5dft_6_0.737
366	P04637_2x0u_2_0.673	1053	P12931_2src_1_0.707
367	P04637_2x0u_1_0.614	1054	P37231_2q6r_1_0.636
368	Q9UM73_4fnw_1_0.76	1055	P29317_5nk4_1_0.662
369	Q06124_6cmr_1_0.811	1056	P49959_3t1i_4_0.699
370	P04637_5lap_1_0.765	1057	P49959_3t1i_2_0.622
371	P10275_1xow_1_0.709	1058	P04629_5i8a_1_0.775
372	P10275_1xow_2_0.691	1059	P03372_4iu7_2_0.679
373	Q13451_4dri_1_0.718	1060	Q9UIF8_4ir5_2_0.676
374	P19793_3nsp_1_0.696	1061	Q9UIF8_4ir5_1_0.738
375	P19793_3nsp_2_0.608	1062	P12931_2bdj_1_0.774
376	P00519_3qri_3_0.757	1063	P51114_3kuf_1_0.786
377	P00519_3qri_1_0.772	1064	Q6P2Q9_4jkf_1_0.686
378	P03372_2bj4_1_0.708	1065	P61586_1kmq_1_0.892
379	P37231_3v9t_1_0.758	1066	Q07157_3tsw_3_0.699
380	Q15047_6bhi_1_0.819	1067	P14210_5ct3_1_0.781
381	Q92793_5ktu_1_0.838	1068	P00519_2hz0_1_0.832
382	Q92793_5ktu_2_0.733	1069	P08581_3u6i_1_0.787
383	P63000_2p2l_3_0.67	1070	Q04771_5s7v_1_0.603
384	Q9H7Z6_4dnc_1_0.775	1071	Q02750_4u80_4_0.609
385	Q9H7Z6_4dnc_3_0.677	1072	A5YKK6_4crv_1_0.75
386	P10275_2q7l_1_0.793	1073	O60674_4d0x_1_0.823
387	P10275_2q7l_2_0.748	1074	P37231_4a4v_2_0.64
388	P61586_3lwn_3_0.754	1075	O60674_3tjc_1_0.621
389	Q9NRY4_3fk2_2_0.721	1076	P68400_5h8b_2_0.704
390	Q02750_3zlx_1_0.729	1077	P37231_2g0g_2_0.714

391	P10275_1t5z_1_0.805	1078	P37231_2g0g_1_0.77
392	P10275_1t5z_2_0.687	1079	P25440_5o3g_1_0.728
393	P04637_2bim_5_0.644	1080	Q16288_4ymj_1_0.823
394	P01112_1gnr_1_0.706	1081	Q16288_4ymj_2_0.623
395	P31749_3qkk_1_0.818	1082	O60674_3iok_1_0.784
396	P01112_221p_1_0.629	1083	O60674_3iok_2_0.778
397	P68400_3owl_1_0.717	1084	P63096_3ums_1_0.742
398	O00571_4pxa_1_0.687	1085	P27797_3pos_2_0.839
399	Q9UM73_3aox_1_0.712	1086	P27797_3pos_1_0.803
400	P01112_3lbn_1_0.707	1087	P27797_3pos_3_0.779
401	Q02750_3wig_1_0.825	1088	P04629_5kmo_1_0.844
402	P00533_4lqm_1_0.705	1089	P22455_4tyg_4_0.618
403	P01112_1nvx_1_0.793	1090	P37231_3vjh_1_0.776
404	P04629_5jfs_1_0.822	1091	P12830_4zte_3_0.613
405	P00533_5xgn_1_0.688	1092	P37231_3fur_1_0.812
406	P03372_5fqr_1_0.852	1093	P09211_5ddl_1_0.62
407	P03372_5tmt_1_0.666	1094	P09211_5ddl_2_0.626
408	P03372_5tmt_4_0.632	1095	P00519_2fo0_2_0.705
409	P15056_4wo5_2_0.656	1096	Q9NZJ5_4x7k_1_0.88
410	Q04771_5s7e_3_0.645	1097	O60674_5aep_1_0.845
411	Q04771_5s7e_2_0.671	1098	O60674_5aep_2_0.706
412	P01112_4g0n_1_0.66	1099	P81274_3sf4_17_0.665
413	Q9UM73_5a9u_1_0.627	1100	P81274_3sf4_1_0.731
414	P03372_5tld_1_0.605	1101	O75581_4a0p_2_0.75
415	P08581_4r1y_1_0.608	1102	P23458_5l04_1_0.73
416	O14980_3gb8_1_0.663	1103	P23458_5l04_4_0.663
417	P37231_3gbk_1_0.74	1104	P43405_4xg3_1_0.645
418	P37231_3gbk_2_0.771	1105	P43405_4xg3_2_0.678
419	P11142_4h5n_1_0.69	1106	O43791_3ivq_1_0.681

420	Q06187_5fbn_2_0.606	1107	P61586_3msx_2_0.636
421	Q06187_5fbn_1_0.63	1108	Q04771_5s7b_2_0.637
422	P01112_1lf5_1_0.636	1109	P04629_5jfx_1_0.867
423	P04637_5mf7_5_0.671	1110	Q9UIF8_5dyx_2_0.628
424	P04637_5mf7_1_0.655	1111	Q9UIF8_5dyx_1_0.856
425	P46100_3qln_9_0.658	1112	Q9UIF8_5pc8_2_0.628
426	P46100_3qln_5_0.643	1113	Q9UIF8_5pc8_1_0.875
427	P10275_2ax7_2_0.743	1114	P08581_3u6h_1_0.748
428	Q04771_3mtf_1_0.67	1115	Q04771_5s83_4_0.738
429	Q04771_3mtf_2_0.694	1116	Q04771_5s83_5_0.66
430	Q9H7Z6_2pq8_1_0.747	1117	Q04771_5s7f_4_0.672
431	Q9H7Z6_2pq8_2_0.687	1118	P04626_5my6_3_0.627
432	P00533_5j9y_2_0.611	1119	P04626_5my6_1_0.622
433	P00533_5ugc_1_0.669	1120	Q9UIQ6_5c97_1_0.738
434	P42336_4l1b_3_0.771	1121	P31751_1o6k_1_0.755
435	P37231_5dv3_1_0.844	1122	P36897_5e8u_1_0.821
436	P03372_2qa8_2_0.695	1123	Q9UIF8_5pbo_2_0.676
437	P09211_5dcg_8_0.685	1124	Q9UIF8_5pbo_1_0.815
438	P09211_5dcg_3_0.609	1125	O43791_3ivv_2_0.637
439	Q15796_1khx_1_0.638	1126	Q06787_4ova_5_0.724
440	P00533_4i20_1_0.745	1127	Q06787_4ova_7_0.622
441	P04637_4ijt_1_0.836	1128	Q86U86_5hrv_1_0.822
442	P00533_5hic_1_0.859	1129	Q9H7Z6_2giv_2_0.74
443	Q02750_4an9_1_0.75	1130	P25054_3nmz_4_0.69
444	P03372_5kce_3_0.745	1131	Q16531_4e5z_1_0.717
445	P03372_5kce_2_0.651	1132	O60674_4aqc_4_0.646
446	EGFR_P00533_2gs6_B_4-8_EIYGE	1133	P49902_2xcx_2_0.681
447	P01112_2c5l_2_0.616	1134	O60674_5ut1_2_0.656

448	P01112_2c5l_3_0.614	1135	Q04771_3oom_1_0.8
449	P01112_2c5l_1_0.811	1136	P08581_3zxz_2_0.809
450	P04637_2vuk_1_0.625	1137	Q13485_1ygs_1_0.666
451	Q14145_5wfv_1_0.712	1138	P37231_4ema_2_0.713
452	Q9BZK7_4lg9_1_0.67	1139	Q9UKU7_1rx0_4_0.832
453	O60674_4e6q_2_0.888	1140	P09211_1md4_1_0.722
454	O60674_4e6q_1_0.868	1141	Q14145_4cxi_2_0.809
455	Q9BYW2_5jle_1_0.831	1142	P68400_3pe2_1_0.709
456	P61586_3msx_1_0.704	1143	P63096_2g83_1_0.661
457	P10721_4hvs_1_0.893	1144	O60674_2b7a_3_0.68
458	P03372_2ouz_1_0.682	1145	Q06187_4otr_2_0.633
459	P15056_4ksp_1_0.64	1146	Q06187_4otr_1_0.744
460	Q9UM73_4clj_1_0.63	1147	P23458_4e4n_2_0.636
461	Q14145_4l7d_3_0.662	1148	Q14145_4iqk_1_0.67
462	P08476_2p6a_11_0.782	1149	O60674_5wim_1_0.748
463	P21802_2pvf_1_0.718	1150	P07954_5d6b_5_0.657
464	P09211_2a2s_1_0.607	1151	O60674_3tjd_4_0.612
465	P09211_2a2s_2_0.688	1152	P68400_3nga_2_0.756
466	O43791_3hqi_3_0.707	1153	P68400_3nga_1_0.835
467	P01116_4wa7_1_0.901	1154	Q86U86_5fh7_1_0.887
468	P37231_3cwd_1_0.752	1155	Q14145_4l7b_3_0.766
469	P00533_4zjv_4_0.64	1156	P11309_4alu_2_0.692
470	P43405_5lmb_1_0.624	1157	P11309_4alu_3_0.617
471	O00571_5e7i_1_0.658	1158	O60674_6bss_1_0.879
472	P00533_3bel_1_0.851	1159	P29317_5ia0_4_0.703
473	P01112_2ce2_1_0.646	1160	Q9UIF8_3q2f_2_0.814
474	Q04771_5s7f_2_0.622	1161	Q9UIF8_3q2f_1_0.828
475	Q04771_5s7f_3_0.796	1162	Q04771_5s7s_4_0.643
476	P61586_3lwn_1_0.764	1163	Q04771_5s7s_3_0.775

477	IDH1_O75874_5k11_A_501_NDP	1164	P31751_1o6l_1_0.787
478	P08581_4eev_1_0.791	1165	P35222_1g3j_1_0.694
479	P10275_4oiu_1_0.811	1166	P03372_4ivw_4_0.653
480	HRAS_P01112_4dlr_A_203_DTU	1167	Q86YC2_2w18_1_0.643
481	O60674_5l3a_1_0.749	1168	P61586_5ez6_1_0.676
482	P04626_3pp0_1_0.854	1169	Q16531_4a08_2_0.674
483	P04626_3pp0_2_0.754	1170	Q9UM73_2yjr_2_0.611
484	P00533_4i24_1_0.742	1171	P09211_4pgt_1_0.629
485	P01116_6bof_4_0.7	1172	P09211_3ie3_2_0.639
486	P01116_6bof_1_0.812	1173	P23458_4e5w_4_0.688
487	P63000_4gzl_1_0.693	1174	Q14145_4l7b_5_0.622
488	P01112_121p_1_0.822	1175	Q16531_3i7n_1_0.836
489	P03372_4zn9_2_0.601	1176	P00519_3ue4_8_0.62
490	P03372_5dkb_2_0.62	1177	P08253_1ck7_1_0.784
491	P03372_5dkb_1_0.63	1178	P08253_1ck7_2_0.737
492	P25440_2e3k_1_0.864	1179	P03372_1xpc_3_0.686
493	P25440_2e3k_4_0.619	1180	P11362_3rhx_1_0.652
494	Q9UIF8_5pe0_2_0.617	1181	P11362_3rhx_3_0.645
495	Q9UIF8_5pe0_1_0.855	1182	Q14145_5dad_1_0.686
496	P42345_4jsv_1_0.643	1183	Q04771_5s7r_5_0.682
497	P42345_4jsv_3_0.641	1184	P25440_2e3k_2_0.866
498	P01112_3rs7_1_0.676	1185	Q86X55_5dwq_1_0.664
499	P01116_4pzy_1_0.669	1186	Q6P2Q9_4jkc_4_0.704
500	P23458_4ivc_2_0.647	1187	Q6P2Q9_4jkc_2_0.654
501	P23458_4ivc_1_0.701	1188	P34896_1bj4_1_0.744
502	P10275_1t65_1_0.828	1189	P08581_4dei_1_0.729
503	P10275_1xj7_2_0.708	1190	P03372_5dkb_4_0.757
504	P10275_1xj7_1_0.78	1191	O60674_5i4n_1_0.869
505	Q9H7Z6_5j8c_1_0.798	1192	Q16531_2b5n_1_0.614

506	P00533_5edq_1_0.729	1193	Q86U86_5e7d_1_0.753
507	P61586_5fr2_1_0.678	1194	Q04771_5s7z_2_0.636
508	P61586_5fr2_3_0.608	1195	Q07157_3tsw_4_0.711
509	P03372_3uud_1_0.631	1196	Q16531_4a11_8_0.61
510	Q9UM73_4ctb_1_0.605	1197	Q16531_4a11_9_0.621
511	P10275_2ao6_2_0.666	1198	P68400_3h30_1_0.767
512	P21802_2q0b_3_0.751	1199	P43405_4fz7_1_0.796
513	P21802_2q0b_1_0.766	1200	O00255_5db3_1_0.689
514	Q13131_4rer_1_0.705	1201	O00255_5db3_2_0.614
515	P10275_2pip_1_0.902	1202	Q04771_5s7e_4_0.651
516	P01112_5wpl_3_0.646	1203	Q8IWS0_4nn2_4_0.605
517	P01112_5wpl_1_0.764	1204	Q6P2Q9_4jkc_5_0.693
518	P00533_4rj8_1_0.686	1205	Q9UM73_4ccu_1_0.637
519	Q14145_3vnh_1_0.698	1206	P29466_2hbr_1_0.733
520	P03372_2ayr_1_0.768	1207	P01116_5vpz_2_0.88
521	O60674_3tjd_3_0.636	1208	P07333_2ogv_1_0.728
522	O60674_3tjd_1_0.697	1209	Q07812_4zif_1_0.714
523	RET_P07949_5fm2_A_2012_PP1	1210	O75874_5yfn_2_0.759
524	Q02750_4anb_1_0.784	1211	Q06787_2qnd_6_0.742
525	P01112_2cl7_1_0.774	1212	P68400_3war_1_0.814
526	Q96L73_3ooi_1_0.755	1213	P09211_5l6x_4_0.703
527	Q04771_5s87_4_0.615	1214	P11142_3ldq_1_0.627
528	Q04771_5s87_2_0.626	1215	Q07157_3shw_1_0.633
529	P00533_5ug8_1_0.799	1216	Q07157_3shw_2_0.608
530	Q04771_5s87_1_0.688	1217	Q13451_4jfi_1_0.682
531	Q04771_5s87_5_0.747	1218	P08238_3pry_1_0.742
532	Q02750_3zls_1_0.731	1219	P08238_3pry_4_0.71
533	P01116_5ocg_1_0.653	1220	P08581_3ce3_1_0.622

534	NTRK1_P04629_6d1y_A_801_FQJ	1221	Q16288_3v5q_1_0.827
535	P04637_3d05_2_0.814	1222	Q07812_4s0p_3_0.755
536	P00533_5hg8_2_0.781	1223	Q07812_4s0p_1_0.667
537	P43405_4xg8_1_0.771	1224	P25440_2dvv_1_0.673
538	P43405_4xg8_3_0.644	1225	P31749_3qkm_1_0.842
539	Q9UMX1_1m1l_1_0.689	1226	O60674_2b7a_2_0.626
540	P37231_5hzc_2_0.601	1227	P12931_6ate_1_0.781
541	P37231_5hzc_1_0.601	1228	P08238_3pry_3_0.722
542	P03372_4xi3_2_0.657	1229	P37231_6c5q_1_0.767
543	P01112_1plk_1_0.705	1230	P68400_3nsz_1_0.741
544	O75874_4xrx_1_0.614	1231	P09211_1aqw_1_0.654
545	O75874_4xrx_3_0.614	1232	Q09472_5kj2_2_0.657
546	SMO_Q99835_5l7i_A_1202_VIS	1233	P43405_4wnm_2_0.645
547	Q04771_5s76_1_0.814	1234	P43405_4wnm_1_0.806
548	Q04771_5s76_3_0.651	1235	P11309_4i41_2_0.634
549	P10275_2pit_1_0.902	1236	P11309_4i41_4_0.638
550	Q16531_3i8c_5_0.699	1237	P31751_1o6k_2_0.615
551	P15056_4e26_1_0.84	1238	O15550_3avr_1_0.652
552	P01112_1lf0_1_0.729	1239	O15550_3avr_2_0.886
553	P12931_1ksw_1_0.71	1240	P43354_1ovl_9_0.669
554	P37231_2q5s_2_0.664	1241	P00519_4twp_4_0.649
555	O75874_4umy_1_0.726	1242	P00519_4twp_3_0.613
556	P10275_4oh5_2_0.828	1243	P03372_5dkb_3_0.672
557	P10275_4oh5_1_0.803	1244	Q9NZJ5_4x7l_1_0.866
558	P06400_4elj_1_0.717	1245	P68400_4ub7_1_0.605
559	P00533_2itp_1_0.744	1246	P43405_3tuc_1_0.778
560	P00533_3ika_3_0.691	1247	O00255_5ddc_2_0.734
561	P00533_5em5_1_0.677	1248	O00255_5ddc_6_0.704

562	P31751_2jdr_1_0.656	1249	P40337_4wqo_3_0.738
563	Q02750_3eqg_1_0.805	1250	P43354_1ovl_3_0.679
564	O60674_3tjc_2_0.773	1251	P43354_1ovl_6_0.88
565	O60674_3tjc_3_0.631	1252	P31939_1pkx_1_0.652
566	P61586_3lwn_2_0.731	1253	P31939_1pkx_3_0.696
567	O75874_5yfn_5_0.779	1254	O75874_4i3k_1_0.623
568	P36897_1py5_1_0.763	1255	P36897_5fri_1_0.877
569	P10275_4oj9_1_0.819	1256	Q06124_5x94_1_0.736
570	Q04771_4dym_1_0.832	1257	Q9UM73_2yhv_2_0.631
571	P03372_4xi3_1_0.641	1258	Q15047_3dlm_1_0.671
572	Q06124_4dgx_2_0.72	1259	P21802_3cly_1_0.824
573	P03372_5kcd_1_0.647	1260	Q04771_5s7s_2_0.628
574	Q15047_6bhe_1_0.69	1261	O60674_5ut4_2_0.821
575	P01116_5vq8_3_0.768	1262	Q9UNN5_3qca_3_0.637
576	P10275_2ao6_1_0.677	1263	Q9UNN5_3qca_2_0.611
577	P08476_2b0u_3_0.644	1264	P03372_4iui_2_0.755
578	P08476_2b0u_6_0.623	1265	P00519_2v7a_1_0.819
579	P01116_5whd_5_0.695	1266	Q06124_5xzr_1_0.801
580	P36897_3gxl_1_0.862	1267	P29466_1bmq_1_0.66
581	P00533_4r5s_1_0.649	1268	P29466_1bmq_3_0.601
582	P22681_5hkx_1_0.84	1269	P68400_3q9w_1_0.663
583	Q14145_4n1b_4_0.622	1270	Q9UIF8_5l8t_2_0.616
584	P23458_4ivb_2_0.618	1271	Q9UIF8_5l8t_1_0.797
585	PIK3CA_P42336_5swo_A_701_2ZV	1272	P25440_5ig6_2_0.75
586	ESR1_P03372_5ehj_A_900_5K5	1273	P25440_5ig6_1_0.675
587	Q14145_5who_1_0.676	1274	Q04771_5s7v_4_0.736
588	P21860_3kex_3_0.623	1275	P01112_4nyj_1_0.722
589	P21860_3kex_2_0.609	1276	O95714_3kci_1_0.718

590	P00519_2hz4_3_0.733	1277	P08476_2arp_1_0.739
591	P00519_2hz4_1_0.668	1278	P08581_4deh_1_0.671
592	Q14145_5who_4_0.707	1279	P31749_5kcv_1_0.811
593	P01116_3gft_2_0.674	1280	P68400_3u9c_2_0.674
594	Q13485_1dd1_1_0.735	1281	O00255_6bxy_1_0.83
595	P37231_3r5n_1_0.768	1282	Q9UIF8_5l98_2_0.61
596	Q9H7Z6_2y0m_1_0.854	1283	Q9UIF8_5l98_1_0.623
597	Q9H7Z6_2y0m_3_0.798	1284	P29466_1rww_3_0.732
598	P04629_5jfs_2_0.692	1285	Q06124_4ohe_1_0.686
599	Q16288_3v5q_2_0.683	1286	P37231_5gtn_2_0.641
600	P37231_5azv_1_0.633	1287	P43405_4i0s_1_0.781
601	P21802_2pwl_2_0.827	1288	P42684_3hmi_2_0.649
602	P21802_2pwl_1_0.79	1289	P42336_5xgh_3_0.703
603	P04040_1dgf_13_0.71	1290	O00255_4og6_1_0.868
604	P04040_1dgf_3_0.749	1291	O00255_4og6_2_0.673
605	P01112_1ctq_1_0.856	1292	Q16288_6kzc_1_0.847
606	Q9UM73_2yfx_1_0.626	1293	P31751_2uw9_1_0.778
607	P03372_1xpc_2_0.737	1294	P52333_4v0g_2_0.772
608	P12830_2o72_1_0.634	1295	P49902_2xjd_1_0.654
609	P37231_4ci5_2_0.637	1296	A5YKK6_4cru_1_0.711
610	P00533_4wd5_1_0.749	1297	P03372_4pps_1_0.714
611	P10275_2z4j_1_0.753	1298	Q13451_4tw7_1_0.753
612	P10275_2z4j_2_0.771	1299	Q9UIF8_5pfe_2_0.699
613	IDH1_O75874_5svf_A_502_70P	1300	Q9UIF8_5pfe_1_0.876
614	P37231_2yfe_1_0.636	1301	Q9H7Z6_2giv_1_0.844
615	Q06187_3p08_1_0.798	1302	P81274_3sf4_4_0.676
616	P15056_5jsm_5_0.625	1303	Q7KZI7_3iec_1_0.697
617	P37231_2p4y_1_0.793	1304	P31749_4gv1_1_0.747
618	P03372_4pp6_1_0.66	1305	Q6P2Q9_4jkg_1_0.698

619	P03372_4pp6_2_0.75	1306	Q9UKU7_1rx0_5_0.74
620	Q13451_5div_1_0.695	1307	P08581_3qti_3_0.65
621	P01112_1p2t_1_0.821	1308	P21580_3dkb_3_0.614
622	P10721_1t46_1_0.862	1309	Q9NYV4_4nst_2_0.639
623	MAP2K4_P45985_3alo_E_3-10_DDEMTPOGYA	1310	Q9NYV4_4nst_5_0.606
624	O75874_4umy_7_0.604	1311	P08581_5eyd_2_0.691
625	O75874_4umy_2_0.789	1312	P61586_3lwn_4_0.757
626	Q04771_5s7b_1_0.673	1313	Q9UIF8_5pd5_2_0.64
627	Q14145_4xmb_1_0.677	1314	Q9UIF8_5pd5_1_0.85
628	O60674_4yti_1_0.818	1315	P03372_5tm2_2_0.648
629	Q04771_5s83_1_0.637	1316	Q9UIF8_5e9k_2_0.624
630	P37231_5ugm_2_0.797	1317	Q9UIF8_5e9k_1_0.602
631	P16671_5lgd_1_0.829	1318	P54764_4bk4_2_0.709
632	P11142_3fzh_1_0.715	1319	Q6P2Q9_4jka_2_0.65
633	P03372_5tlv_3_0.723	1320	Q6P2Q9_4jka_1_0.725
634	P03372_5tlv_2_0.705	1321	Q9H7Z6_3toa_1_0.89
635	Q02750_4u80_2_0.852	1322	Q92793_4nyv_2_0.716
636	P08581_5dg5_2_0.624	1323	P43405_4gfg_1_0.702
637	P04629_5kmi_1_0.756	1324	P25440_5xhe_1_0.73
638	Q04771_5s8a_3_0.755	1325	P35968_4ase_1_0.805
639	Q14145_4ifn_1_0.647	1326	P14210_1si5_3_0.626
640	P10275_1t63_1_0.788	1327	P11309_4n6z_2_0.64
641	P10275_1t63_2_0.791	1328	P11309_4n6z_1_0.784
642	Q14145_4l7b_2_0.786	1329	P09211_1md4_2_0.612
643	Q14145_4l7b_1_0.858	1330	P00519_2e2b_1_0.663
644	Q13485_1g88_5_0.605	1331	P25440_5bt5_1_0.853
645	Q92793_3dwy_1_0.803	1332	Q04771_5s82_1_0.631
646	P07949_2ivv_1_0.701	1333	Q04771_5s82_3_0.673

647	P01112_4q21_1_0.785	1334	P36897_5e8x_2_0.615
648	P35968_3b8r_3_0.661	1335	P36897_5e8x_1_0.7
649	P68400_3q9z_3_0.665	1336	P61769_4r9h_2_0.677
650	P68400_3q9z_2_0.693	1337	P46531_2f8y_5_0.864
651	P00519_3ue4_1_0.618	1338	P46531_2f8y_2_0.69
652	P25440_2e3k_5_0.782	1339	O00571_5e7m_2_0.654
653	P25440_2e3k_3_0.671	1340	P08581_5hni_5_0.628
654	P01112_5wpl_5_0.673	1341	P09211_1pgt_2_0.707
655	P01112_5wpl_4_0.666	1342	P68400_3at4_1_0.852
656	Q06124_4ohd_1_0.77	1343	Q92793_5h85_1_0.727
657	O60674_3tjc_4_0.657	1344	Q16832_2wuh_1_0.819
658	P08476_2p6a_6_0.805	1345	P68400_5ku8_2_0.7
659	P63000_1mh1_1_0.783	1346	P21359_3pg7_1_0.674
660	Q09472_5lkt_1_0.716	1347	P21359_3pg7_2_0.607
661	Q09472_5lkt_2_0.616	1348	Q9NR48_3mqm_2_0.687
662	P04629_4pmt_1_0.756	1349	Q9NR48_3mqm_1_0.786
663	Q04771_3mtf_3_0.775	1350	P36897_1rw8_1_0.879
664	P03372_3dt3_2_0.614	1351	Q14145_4cxj_1_0.846
665	P03372_1err_1_0.655	1352	Q9UMX1_4km9_1_0.758
666	Q04771_4c02_1_0.655	1353	P09211_1lbk_2_0.601
667	Q04771_4c02_2_0.822	1354	Q04721_2oo4_3_0.787
668	P37231_4pvu_1_0.655	1355	Q9Y6K1_3llr_1_0.796
669	P37231_4pvu_2_0.674	1356	P01112_1nvu_4_0.767
670	Q9BYW2_5lsy_1_0.666	1357	P21359_3peg_1_0.816
671	P03372_4znw_2_0.624	1358	Q6P2Q9_4jk8_1_0.835
672	P31751_3d0e_4_0.617	1359	O75874_1t09_5_0.772
673	P31751_3d0e_1_0.858	1360	Q96QB1_3kuq_1_0.612
674	P37231_2yfe_2_0.672	1361	Q96QB1_3kuq_3_0.647
675	Q9UPY3_2eb1_2_0.704	1362	P07949_4ckj_1_0.655

676	P68400_3q9y_1_0.656	1363	P43246_3thx_4_0.656
677	PTPN11_Q06124_5ehr_A_601_5OD	1364	P51532_5ea1_2_0.645
678	P03372_5dks_1_0.708	1365	P06401_3g8o_1_0.635
679	Q9UM73_4fnw_3_0.657	1366	O60674_4hge_2_0.717
680	PIK3CA_P42336_5sw8_A_1103_FB1	1367	P35222_2gl7_2_0.659
681	Q9H7Z6_2y0m_2_0.615	1368	P35222_2gl7_3_0.688
682	KDR_P35968_5oyj_A_9-169	1369	P25440_5o3d_1_0.881
683	P61586_3lwn_6_0.625	1370	Q9UIF8_5mge_2_0.669
684	P42336_5swp_3_0.734	1371	Q9UIF8_5mge_1_0.626
685	P01112_4dlx_1_0.73	1372	Q86YC2_3eu7_1_0.633
686	P10275_2axa_1_0.813	1373	P31749_3mvh_1_0.715
687	Q04771_5s7u_2_0.649		

[a] the allosteric site ID indexed in ASD download (http://mdl.shsmu.edu.cn/ASD)

Table S5. Hyperparameters fine-tuning for the ET model.

Batch size	Embedding dimension	No. of layers	No. of attention heads	No. of radial basis functions
32, **64**, 128	64, **128**, 256	**6**, 8	**8**, 10	**32**, 64

Table S6. Performance metrics in test dataset of DeepAlloDriver.

	accuracy	precision	recall	specificity	F1 Score	AUROC
Mean	0.9412	0.9384	0.9433	0.9390	0.9408	0.9747
Std	0.0078	0.0044	0.0126	0.0034	0.0084	0.0047

Table S7. Performance metrics in test dataset of AlloDriver and DeepAlloDriver.

Method	accuracy	precision	recall	specificity	F1 Score	AUROC

DeepAlloDriver	0.9412	0.9384	0.9433	0.9390	0.9408	0.9747
AlloDriver	0.5345	0.5598	0.3333	0.7367	0.4179	0.5435

4. Figures

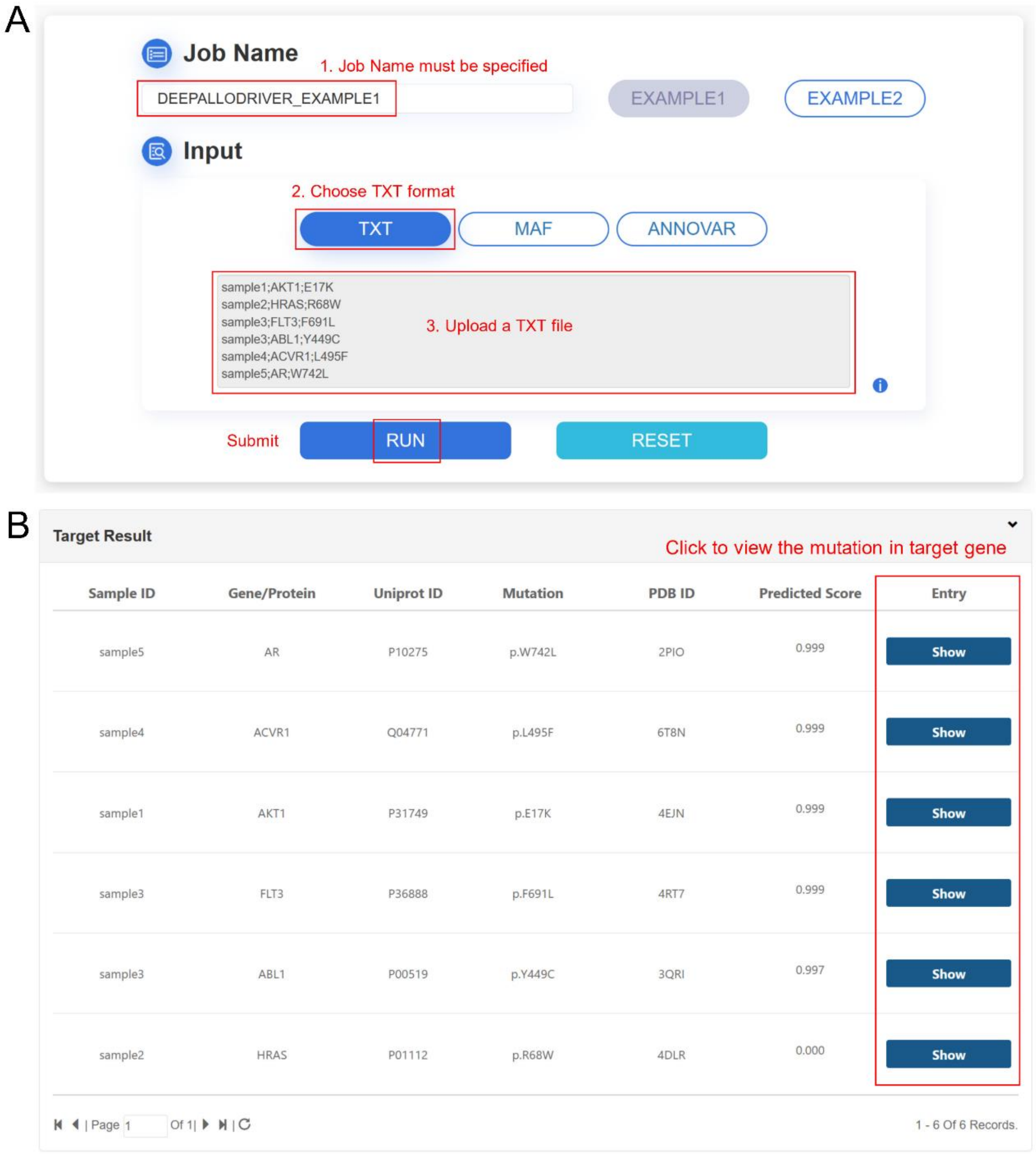

Figure S1. Screenshots of the DeepAlloDriver procedure from Example 1. (**A**) Input page of DeepAlloDriver. (**B**) Output result table of Job.

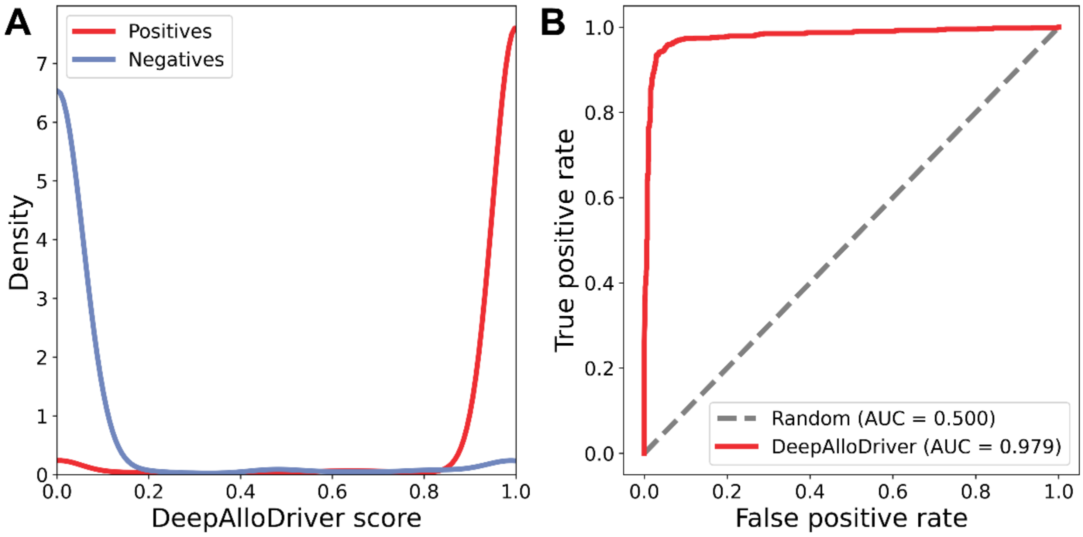

Figure S2. Performances of DeepAlloDriver. (**A**) The DeepAlloDriver score distributions of driver mutations (labelled positives) and non-driver mutations (labelled negatives), and (**B**) the Receiver operating characteristic (ROC) curve of the best model of DeepAlloDriver in predicting driver mutations on the test set.

REFERENCE

1. Tamborero, D., Rubio-Perez, C., Deu-Pons, J., Schroeder, M.P., Vivancos, A., Rovira, A., Tusquets, I., Albanell, J., Rodon, J., Tabernero, J. *et al.* (2018) Cancer Genome Interpreter annotates the biological and clinical relevance of tumor alterations. *Genome Medicine*, **10**, 25.
2. Zhang, J., Lee, D., Dhiman, V., Jiang, P., Xu, J., McGillivray, P., Yang, H., Liu, J., Meyerson, W., Clarke, D. *et al.* (2020) An integrative ENCODE resource for cancer genomics. *Nature Communications*, **11**, 3696.
3. Griffith, M., Spies, N.C., Krysiak, K., McMichael, J.F., Coffman, A.C., Danos, A.M., Ainscough, B.J., Ramirez, C.A., Rieke, D.T., Kujan, L. *et al.* (2017) CIViC is a community knowledgebase for expert crowdsourcing the clinical interpretation of variants in cancer. *Nature Genetics*, **49**, 170-174.
4. Ainscough, B.J., Griffith, M., Coffman, A.C., Wagner, A.H., Kunisaki, J., Choudhary, M.N.K., McMichael, J.F., Fulton, R.S., Wilson, R.K., Griffith, O.L. *et al.* (2016) DoCM: a database of curated mutations in cancer. *Nature Methods*, **13**, 806-807.
5. Chakravarty, D., Gao, J., Phillips, S., Kundra, R., Zhang, H., Wang, J., Rudolph, J.E., Yaeger, R., Soumerai, T., Nissan, M.H. *et al.* (2017) OncoKB: A Precision Oncology Knowledge Base. *JCO Precision Oncology*, 1-16.
6. Huang, L., Fernandes, H., Zia, H., Tavassoli, P., Rennert, H., Pisapia, D., Imielinski, M., Sboner, A., Rubin, M.A., Kluk, M. *et al.* (2016) The cancer precision medicine knowledge base for structured clinical-grade mutations and interpretations. *Journal of the American Medical Informatics Association*, **24**, 513-519.
7. Chang, K., Creighton, C.J., Davis, C., Donehower, L., Drummond, J., Wheeler, D., Ally, A., Balasundaram, M., Birol, I., Butterfield, Y.S.N. *et al.* (2013) The Cancer Genome Atlas Pan-Cancer analysis project. *Nature Genetics*, **45**, 1113-1120.
8. Zhang, J., Bajari, R., Andric, D., Gerthoffert, F., Lepsa, A., Nahal-Bose, H., Stein, L.D. and Ferretti, V. (2019) The International Cancer Genome Consortium Data Portal. *Nature Biotechnology*, **37**, 367-369.
9. Tate, J.G., Bamford, S., Jubb, H.C., Sondka, Z., Beare, D.M., Bindal, N., Boutselakis, H., Cole, C.G., Creatore, C., Dawson, E. *et al.* (2018) COSMIC: the Catalogue Of Somatic Mutations In Cancer. *Nucleic Acids Research*, **47**, D941-D947.
10. Schrodinger, LLC. (2015).
11. Huang, W., Lu, S., Huang, Z., Liu, X., Mou, L., Luo, Y., Zhao, Y., Liu, Y., Chen, Z., Hou, T. *et al.* (2013) Allosite: a method for predicting allosteric sites. *Bioinformatics*, **29**, 2357-2359.
12. Berman, H.M., Westbrook, J., Feng, Z., Gilliland, G., Bhat, T.N., Weissig, H., Shindyalov, I.N. and Bourne, P.E. (2000) The Protein Data Bank. *Nucleic Acids Research*, **28**, 235-242.
13. Dana, J.M., Gutmanas, A., Tyagi, N., Qi, G., O'Donovan, C., Martin, M. and Velankar, S. (2018) SIFTS: updated Structure Integration with Function, Taxonomy and Sequences resource allows 40-fold increase in coverage of structure-based annotations for proteins. *Nucleic Acids Research*, **47**, D482-D489.

14. The UniProt, C. (2021) UniProt: the universal protein knowledgebase in 2021. *Nucleic Acids Research*, **49**, D480-D489.
15. Thölke, P. and De Fabritiis, G. (2022) TorchMD-NET: Equivariant Transformers for Neural Network based Molecular Potentials. 10.48550/arXiv.2202.02541.
16. Vaswani, A., Shazeer, N., Parmar, N., Uszkoreit, J., Jones, L., Gomez, A.N., Kaiser, Ł. and Polosukhin, I. (2017) Attention is all you need. *Advances in neural information processing systems*, **30**.
17. Chakrabarty, B. and Parekh, N. (2016) NAPS: network analysis of protein structures. *Nucleic acids research*, **44**, W375-W382.
